# Removal of fillers and chemical reagents from waste paper for its sustainable use

**DOI:** 10.1007/s11356-025-37133-5

**Published:** 2025-11-07

**Authors:** Marek Kucbel, Helena Raclavská, Jana Růžičková, Michal Šafář, Pavel Kantor, Karolina Slamová, Jarmila Drozdová

**Affiliations:** 1 CEET/ENET Centre, VSB–Technical University of Ostrava, 17. listopadu 15/2172, Ostrava-Poruba, 70800 Czech Republic; 2 Institute of Foreign Languages, VSB–Technical University of Ostrava, 17. listopadu 15/2172, Ostrava-Poruba, 70800 Czech Republic; 3 Department of Mechanical Engineering, Faculty of Technology, Institute of Technology and Business in České Budějovice, Okružní 517/10, České Budějovice, 37001 Czech Republic

**Keywords:** Waste office paper, Cellulose fibres, Precipitated calcium carbonate, Organic pollutants, Health hazards

## Abstract

Waste paper represents a valuable secondary source of cellulose fibres, contributing to the reduction of virgin wood consumption in paper production. To support sustainable development objectives, the integration of natural fibres with synthetic polymers is increasingly explored across industrial sectors. Reliable use of waste paper materials requires baseline data on pollutant content and migration potential. In this study, cellulose fibres were separated using 0.2 M acetic acid (CH_3_COOH) extraction, followed by washing. This method effectively removed precipitated calcium carbonate-based filler from office paper, achieving an extraction efficiency of 86%. A total of 138 compounds were identified in waste paper, originating from: virgin wood (*n* = 31), paper manufacturing and recycling processes (*n* = 19 + 15 fragrance compounds), and printing inks (*n* = 67), with solvents (*n* = 25) forming the largest subgroup. Additional substances were associated with surface treatments and ink formulations. Compound hazard profiles were assessed using the Globally Harmonized System (GHS). The identified substances were, among others, persistent organic pollutants, including benzophenone*,* butylated hydroxytoluene*,* and bis(2-ethylhexyl) phthalate*,* bisphenol A, and bisphenol S*,* while solvents exhibited the highest proportion of hazardous classifications. Following CH_3_COOH extraction, concentrations of hazardous solvents were reduced by 93%, indicating the method’s potential for pollutant mitigation in fibre recovery.

**Supplementary Information:**

The online version contains supplementary material available at 10.1007/s11356-025-37133-5.

## Introduction

In the Czech Republic, 40–50 kg of waste paper is produced per person/year. According to Milbrandt et al. (2024), the largest share of the waste paper is achieved by cardboard (44%); compostable paper, food-soiled paper products, towels, and napkins (21%); other papers, books and aseptic containers (18%); newspapers (6%); high-grade office paper (6%); and journals (5%). Increasing paper consumption produces more waste paper. Thus, the extraction of pulp from paper waste can reduce negative impacts on forest ecosystems (Hanafiah et al. 2019). Waste paper is a significant raw material whose value can be efficiently increased according to the rules of the circular economy. While the largest proportion of recycled cellulose fibres is still used for paper production and products (for example, pressed fibres), a sharp increase has been seen mainly in biodegradable food transport packaging (Andrade et al. 2022) based on plant fibre/plastic composites (PPCs). The potential safety risk of plant fibres is the crucial distinction between PPCs and common plastic materials (Zhang And Weng 2021). Despite the rapid development of technologies for processing cellulose fibres and cellulose nanocrystals (CNs) in the packaging industry, there are some doubts about the safety of these products made from waste cellulose fibres in connection with releasing volatile migrants from packaging or printing (An et al. 2024). However, systematic research on migration methods and safety assessment is still insufficient, and further studies are needed regarding the main safety risks and migration patterns.

For further utilization of cellulose fibres, their quality is a determining factor. Standard technologies for recovering cellulose fibres from waste paper include disintegration using sodium hydroxide (NaOH), followed by coarse screening of mechanical impurities and a deinking process involving flotation and washing. Flotation enables the removal of fillers and inks, while washing eliminates residual microparticles from the suspension. These procedures may be modified through enzymatic treatment (Singh et al. 2020) and thermomechanical dispersion. However, when removing fillers, especially precipitated calcium carbonate (PCC), flotation may not be sufficiently effective. PCC particles are typically smaller than 5 µm, which is below the optimal separation range of flotation. In addition, PCC is hydrophilic and therefore poorly adheres to air bubbles; this limitation, however, can be overcome with the use of collectors (Hubbe And Gill 2016).

The choice of chemical reagent depends on the intended application of the cellulose fibres. Strong acids such as hydrochloric acid (HCl) cause hydrolysis of glycosidic bonds within cellulose, leading to fibre shortening and reduced crystallinity, particularly in amorphous regions (Habibi et al. 2010). In contrast, low concentrations of acetic acid do not affect the crystalline structure of cellulose, nor do they result in acetylation (Hu et al. 2018). Acetic acid was therefore selected for its gentle effect on the fibres and its selective reactivity with PCC. The reaction yields soluble calcium acetate, which can be easily removed by washing. This approach minimizes both the risk of cellulose degradation and the technological and environmental concerns associated with the use of aggressive mineral acids.

In addition to the standard recycling of waste paper, the subject of interest is increasing its value in the form of new products, such as the production of cellulose nanocrystals (CNs). Many reactive chemical groups are present on the surface of CNs, allowing physical adsorption, surface modification, and chemical vapour deposition. This ensures the use of CNs in a wide range of applications, both in their original state and based on other chemical modifications (Rashid et al. 2023). These properties make it possible for them to be applied extensively in the industry, including construction, cuisine, telecommunications, the automotive industry, medication, the cosmetic industry, and the production of carbon nanotubes, representing another rod-shaped nanomaterial with excellent mechanical and electrical conductivity performance (Yang et al. 2022; Mohammad Firman et al. 2023). In the environment, CNs are used as the hybrid bio-sponge sorbent with magnetic bimetallic Fe_3_O_4_@TiO_2_ for coloured water treatment and oil–water separation (Assanvo et al. 2023). Moreover, CNs and CNFs have been widely investigated for wastewater remediation due to their high surface area and the possibility of changing the surface chemistry. Functionalized nanocellulose has shown excellent sorption capacity for heavy metals (e.g. Pb^2+^, Cr^6+^, and Cd^2+^), dyes, pesticides, and oil residues. Forms such as aerogels, membranes, and hydrogels have been applied in various water purification strategies (Abdelhamid 2024; Yang et al. 2024).

There is increasing use of environmentally sustainable products based on the use of cellulose fibres in the construction industry, where bio-compound (natural fibre) has the function of reinforcement in polymer matrix composites as it increases the mechanical strength of composites (Uppal et al. 2022). Cellulose fibres have recently been used in soil protection against erosion (Ishak et al. 2021) or in the production of hydrogels, which have a hydrophilic structure with the ability to retain large amounts of water within their three-dimensional networks (Ioelovich 2021). The hydrogels have a more comprehensive application; they can be used in biomedicine, hygiene, pharmaceutics, food processing, chemistry, physical chemistry, water purification, agriculture, and other areas.

Cellulose fibres obtained from waste paper may be contaminated with additives that are used in paper production or are part of printing inks and adhesives. A higher degree of evaluation of cellulose fibres requires their higher purity (Pivnenko et al. 2015). Up to 348 compounds that may be present in paper material are listed in the literature (Pivnenko et al. 2016). Up to 157 potentially hazardous compounds may be present in paper material. A total of 133 chemical substances have been assigned to the printing industry, the majority of which are solvents and polymer resins used in inks, pigments, and dyes. Chemical compounds that cannot be assigned to either paper production or the printing industry could potentially be by-products or contaminants introduced into the production cycle through recycled paper (Pivnenko et al. 2016).

Information on the occurrence of pollutants in waste paper should be crucial in deciding how to further apply the cellulose fibres used, especially considering their potential use for the food packaging industry. There is a lack of published data on the ability of weak organic acids to target additives used in paper production. Our findings are supported by the results of Lee et al. (2011), Singh et al. (2020), and Itkor et al. (2024), all of which confirm that mild acids alone are capable of significantly reducing additive content in separated cellulose fibres.

The article aims to identify compounds that occur in waste paper and come from virgin wood (e.g. pesticides) or paper production technology, as well as the use of printing inks. It can provide the missing information on the potential risks involved in using cellulose fibres from waste paper. The second objective was to assess the impact of removing calcium carbonate on the quality of the cellulose fibres from the point of view of their further potential use. The originality of this research lies in the systematic identification of a broad spectrum of organic pollutants present in waste paper and the verification of an extraction method capable of removing hazardous compounds effectively, without deteriorating the quality of cellulose fibres. Through the application of TD-GC/MS and acetic acid treatment, the study uncovers previously undetectable compounds embedded within the paper matrix. It confirms their origin from printing inks and virgin wood. This approach demonstrates that waste paper, particularly office paper, can be transformed into a highly pure cellulose source suitable for further technological use. The work contributes new evidence supporting sustainable paper decontamination and offers an analytically supported method to improve recyclability and safety in circular material flows.

## Materials and methods

### Materials and their pre-treatment for further analysis

Separated samples of waste paper (office paper, magazines, and cardboard) were obtained from Smolo Co., a company specializing in waste management. Three samples collected at 1-month intervals were analysed.

Three office paper samples were collected at monthly intervals from the shredding facility operated by Archivace Skartace Co. in Ostrava, which provides paper shredding services within the city (Moravian-Silesian Region, Czech Republic). This company processes approximately 540 tonnes of office paper annually. During each sampling session, 100 kg of waste office paper was collected and reduced on-site to a final weight of 10 kg. The sample was then transported to the laboratory, where it was quartered to the required weight of 1 kg (Hennebert And Beggio 2021). These prepared samples were used for chemical analysis and cellulose fibre separation.

Samples of magazines, cardboard, and mixed paper were obtained from the sorting line operated by Smolo Co. The sorting line produces approximately 150 kg/h of cardboard, 7.5 kg/h of magazine paper, and 30 kg/h of mixed paper. On a single day, 120 kg of cardboard, 24 kg of mixed paper, and 10 kg of magazine paper were collected. Sampling was conducted three times. After collection, cardboard and mixed paper samples were shredded to obtain 10 kg portions. The magazine paper was not quartered. The chemical analysis results reported represent the average values obtained from three independent samples.

Waste paper samples were cut into smaller pieces (approximately 1 cm). To decompose carbonates, a mixture of 20 g of office paper and 500 mL of 0.2 M CH₃COOH was prepared. The use of acetic acid does not represent a novel extraction method; however, it is well documented that this approach enables the recovery of cellulosic fibres with minimal damage, specifically, without significant loss of crystallinity or cleavage of glycosidic bonds. Importantly, acetic acid allows for the simultaneous removal of both PCC and organic additives, without compromising the cellulose structure. In contrast, traditional acid or alkaline treatments (e.g. NaOH and HCl) often exhibit limitations in selectively removing both fillers and additives while preserving fibre integrity.

The sample of waste paper was disintegrated for 1 min using the Vorwerk Thermomix TM6 (Vorwerk Engineering, Wuppertal, Germany) and then separated in a centrifuge (Beckman Avanti JXN-26, Beckman Coulter, Brea, California) at 8000 rpm. The acid–base reaction of the solution was determined according to ISO 10523 (International Organization for Standardization 2008). Calcium ion concentration (Ca^2^⁺, mg/L) was analysed following ISO 6058 (International Organization for Standardization 1984). The cellulose sample was subsequently rinsed with 500 mL of deionized water (once or twice). The selection of a suitable extraction agent and its concentration (HCl, H₃PO₄, and CH₃COOH) was based on the studies by Phipps and Lorusso (2001) and Kim and Kim (2018).

### Methods

The ASTM E1755-01(2020) “Standard Test Method for Ash in Biomass” (oxidation at 575 ± 25 °C) was applied (ASTM International 2020). This method is suitable for determining ash content in materials such as office waste, boxboard, and newsprint. Moisture content was determined according to ISO 18134-3:2023 (International Organization for Standardization 2023). The calcium (Ca) concentration in both embossed cellulose and waste paper was measured using the US EPA Method 6200, “Field Portable X-ray Fluorescence Spectroscopy for the Determination of Elemental Concentrations in Soil and Sediments,” employing the Innov-X Delta Professional analyzer (Olympus Innov-X, USA).

Identifying minerals in ash and the character of cellulose fibres was performed using the auto-emission scanning electron microscope FEI Quanta-650 FEG (manufactured by FEI Co., Hillsboro, USA). The mineralogical phase composition of fillers was analysed by X-ray diffraction (Bruker Advance D8 X-ray diffractometer).

The chemical composition and crystallinity of the separated cellulose were analysed using a Fourier transform infrared (FTIR) spectrometer (FT-IR Nicolet 6700, Thermo Scientific). Measurements were conducted using the attenuated total reflectance (ATR) technique across a spectral range of 4000 to 525 cm⁻^1^, with a resolution of 4 cm⁻^1^. Each sample was scanned 64 times, and the resulting spectra were compared to assess structural differences and compositional features.

The TD-GC/MS method (Gerstel, Mülheim an der Ruhr, Germany) was used for the identification of organic compounds in the feedstock. A sample of 300 µg of paper/cardboard/magazine was placed into a glass sampling tube along with an internal standard (1,3,5-tri-tert-butylbenzene). Thermal desorption was performed using a unit (Gerstel, Mülheim an der Ruhr, Germany) in the temperature range 50 to 300 °C for 5 min, with a heating rate of 60 °C/min. Volatilized organic compounds were concentrated in a cooled injection system (CIS) at − 10 °C and subsequently separated on a non-polar HP5 ms column (60 m × 0.25 mm × 0.25 µm) under a temperature programme: 40 °C (2 min) to 310 °C (10 min), at a rate of 10 °C/min. Identification and quantification of organic compounds were performed using a mass spectrometer (Agilent 5977 B, Santa Clara, USA) within a scan range of m/z 50–650, using external calibration with certified standards. GC/MS quantification of compounds was performed by external standards with the addition of 1 µl of internal standard (1,3,5-tri-tert-butylbenzene) by the calibration curve method. The construction of calibration curves was carried out by the program Mass Hunter-MS Quantification.

### Processing of results from TD-GC/MS

A comprehensive search of the literature, PubChem databases, and additional online sources was conducted to identify the origin of the detected compounds. This included information from the “European Printing Ink Association (EuPIA), Inventory List – Version January 2011, ” which catalogues packaging ink raw materials applied to the non-food contact surfaces of food packaging. The potential hazards of the identified compounds were assessed according to the “Globally Harmonized System of Classification and Labelling of Chemicals” (GHS Rev. 10, 2023), a guideline for the classification and labelling of hazardous substances. To characterize the broadest possible range of identified chemical compounds, the use of GHS hazard categories was selected as the only viable and consistent method for communicating their potential risks across international datasets, since GHS-based databases contain information on hundreds of thousands of substances, including many that are not registered in the EU. This approach allowed us to retrieve relevant hazard data for a large number of organic compounds.

For each compound, hazard information from the PubChem database was consulted, with emphasis on the GHS hazard statements: health risks, environmental risks, and irritants—the latter being considered part of health risks. Classification into hazard categories was performed using the comprehensive category system provided by the United Nations Economic Commission for Europe. In cases where a compound was associated with risks in all three categories, only one hazard class was reported, following a predefined prioritization: health risks > environmental risks > irritants.

## Results

### Paper composition

A wide variety of chemicals are used in paper production, selected to optimize paper properties and maximize production efficiency while complying with environmental regulations. The specific grade of paper being produced also influences chemical selection. One strategy for promoting environmental sustainability in the paper industry involves creating a novel composite material by combining paper with polyester, including the potential use of waste products (Sheeju Selva Roji et al. 2024). The incorporation of short-cut polyester fibres (polyethylene terephthalate) enhances the wet strength of the paper, enabling its use in high-humidity environments without disintegration. This polyester addition facilitates the production of fibre-reinforced paper, commonly used in cardboard boxes and paper bags. In order to improve adhesion between cellulose and synthetic fibres, a vinyl-acrylic binder is applied. Furthermore, when paper is used as a packaging material, its hydrophobic properties are of particular importance. These can be achieved through surface treatment with silane-modified starch (Manoharan et al. 2021; Majka et al. 2023).

The properties of paper are also influenced by the presence of residual extractives and adhesive substances known as “stickies.” In addition to the primary components—hemicellulose, cellulose, and lignin—wood contains a range of extractives. The proportion of these major components varies depending on the paper type. For office paper, cellulose ranges from 61.8% to 79.2%, hemicellulose from 3.5% to 12.6%, and lignin from 2.0% to 9.2% (De Oliveira et al. 2023). In cardboard, the cellulose content is lower (56.9%), while lignin is higher, up to 17.8%, and hemicellulose accounts for approximately 10.7% (Vukoje and Rožić 2018). Extractives typically represent 2–5% of softwood composition and include waxes, fats, terpenes and terpenoids, fatty acids (e.g. palmitic and stearic acids), monosaccharides, alkaloids, and phenolic compounds such as simple phenols, lignans, flavonoids, tannins, and stilbenes (N’Guessan et al. 2023). The presence of extractives is known to diminish pulp quality. These compounds are soluble in various neutral solvents. Depending on the pulping technology used, up to 85% of extractives may be removed during processing (Lehr et al. 2021).

Organic sticky contaminants affect both the papermaking process and the quality of the final paper product. These contaminants—including polyvinyl acetate polymers, styrene-butadiene rubber, polyamines, and paraffin waxes derived from wood extractives—vary depending on the paper grade (Wang et al. 2023). They originate primarily from the addition of coating binders during production, but may also come from recycled paper containing printing inks, such as styrene-butadiene rubber and polyvinyl acrylate.

Odorous compounds can arise during both the technological processing of paper and its ageing. The papermaking process occurs in a moist and temperature-sufficient environment rich in nutrients. The increased use of recycled paper contributes to these conditions, promoting microbial development. Recycled fibres often contain residues from sizing agents, coatings, starches, polymers, and adhesives, all of which are nutrient-rich (Czerny And Buettner 2009). Anaerobic decomposition of these compounds leads to the formation of volatile fatty acids, which are major contributors to unpleasant odours. To suppress microbial growth and mitigate odour, biocidal agents are introduced, including essential oils that not only provide fragrance but also possess antibacterial properties.

Odour-inducing compounds may be released as a result of microbial degradation of lignin and the autooxidation of cardboard. These include odour-active aldehydes such as hexanal, heptanal, octanal, and nonanal (Czerny And Buettner 2009). Paper degradation caused by natural ageing leads to the formation of low molecular weight compounds, including, among others, formic, acetic, lactic, propionic, and levulinic acids (Jablonsky et al. 2012), which also contribute to odour emissions. Another source of odour precursors may be unsaturated lipids present in cardboard used for food packaging. Ultraviolet (UV) curable inks containing photoinitiators are commonly applied in the printing of cellulose-based packaging materials (Pugh And Guthrie 2000), influencing the rate and extent of lipid oxidation.

Fragrances are incorporated to prevent the spread of unpleasant odours associated with paper ageing, food storage, or during the manufacturing of scented paper products. Scent can be added by embedding solid or liquid fragrances during the papermaking process, by introducing fragrance-loaded nano/microspheres directly into the pulp, or through microencapsulation, whereby encapsulated compounds are adsorbed onto the paper surface (Rungwasantisuk And Raibhu 2020). Common encapsulated fragrance agents include lavender essential oil, vanillin, citronella oil, and orange oil. These are delivered using carriers such as chitosan, carboxymethylcellulose, or the triblock copolymer polyethylene oxide–polypropylene glycol–polyethylene oxide (PEO–PPO–PEO). Recently, encapsulated fragrances with antibacterial properties have also been introduced into paper products (Perinelli et al. 2020).

#### Fillers

The most common fillers in paper production are minerals, either of natural origin or produced synthetically. Calcite is widely used as a filler, available both in its natural form and as synthetic precipitated calcium carbonate (PCC). Fillers are incorporated into cellulose fibres at the early stage of the papermaking process. The quantity of filler used depends on the intended application of the paper. For office paper, filler content typically ranges between 5 and 30%. For PCC specifically, the recommended maximum concentration is 20–25%, depending on the paper grade (Dölle 2021). Notably, certain products, such as Kleenex, contain no fillers at all. When fillers are used as substitutes for cellulose fibres, several advantages are commonly cited: reduced consumption of natural fibres, lower drying energy costs, and modified paper properties. Incorporating fillers enhances brightness and opacity due to their particle size characteristics. Depending on the polymorphous phase contained in PCC, fillers can affect friction and pore size (Hubbe And Gill 2016), as well as surface smoothness and ink absorption in papermaking (Jimoh et al. 2018). Beyond their role as fillers, PCC is also utilized as a coating material. For effective performance in paper production, calcite should exhibit a highly uniform particle size distribution. At least 75 wt.% of particles should have a diameter below 1 μm for coatings, or below 5 μm when used as fillers (Dhar et al. 2020). The particle size of PCC can be reduced through the addition of organic compounds, such as ethylene glycols, which also promote the formation of highly aggregated crystals (Konopacka-Łyskawa et al. 2017). PCC modification is carried out using chemical agents like chitosan, acetic acid, carboxymethyl cellulose (CMC), and alum (Al_2_(SO_4_)_3_·nH_2_O), improving physical characteristics (Jimoh et al. 2018; Ghosh et al. 2020). Alterations in crystal morphology have also been achieved using ammonium carbamate and urea (Liendo et al. 2022). The use of CMC and polyaluminium chloride (PAC) for encapsulating PCC fillers has been shown to enhance paper properties, particularly brightness and opacity (Mousavipazhouh et al. 2018).

**Fig. 1:**
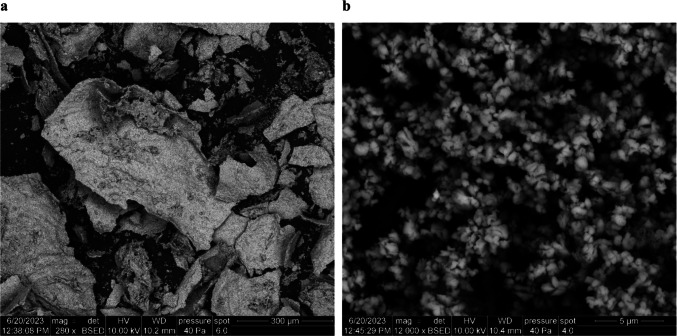
Ash (dicalcium silicate) after combustion of journals at 815 °C (**a**); ash (micro-calcite) after combustion of office paper (**b**)

Differences in mineralogical composition and filler content are presented in Table 1, with filler proportions corresponding to the measured ash content (Fig. 1). All three forms of calcium carbonate are employed in office paper production. In journal-grade papers, synthetic dicalcium silicate derived from fly ash has emerged as a significant filler component, recently introduced to the paper industry, particularly for high-quality applications (Song et al. 2018; Qiu et al. 2020).

**Table 1:** Mineral composition of fillers in different kinds of used paper (weight %) determined by the X-ray diffraction method

Mineral phase	Chemical formula	Office paper	Journals	Cardboard	Mixture
		wt. %			
Anhydrite	CaSO_4_		0.40 ± 0.18	0.12 ± 0.04	
C2Sα	Polymorphic dicalcium silicate (2CaO*SiO_2_)	0.63 ± 0.28	10.25 ± 3.57		0.33 ± 0.15
Portlandite	Ca(OH)_2_		6.81 ± 3.23		1.09 ± 0.42
Lime	CaO	2.56 ± 0.87	0.88 ± 0.36	0.24 ± 0.11	
Calcite	CaCO_3_–trigonal	9.08 ± 2.55	11.30 ± 13.20	10.19 ± 3.56	6.88 ± 2.19
Vaterite	CaCO_3_–hexagonal	1.09 ± 0.22			3.40 ± 0.78
Aragonite	CaCO_3_–orthorhombic	1.11 ± 0.38			
Talk	Mg_3_Si_4_O_10_(OH)_2_			0.05 ± 0.02	
Quartz	SiO_2_			0.27 ± 0.14	
Ash		14.47 ± 2.87	29.05 ± 10.87	10.87 ± 2.26	11.70 ± 3.37

#### Printing inks

Modern printing inks typically consist of pigments (5–30%); binders (15–50%), which may include oils, resins, or various types of varnishes; solvents (15–65%); and excipients (< 10%) such as drying agents, chelating compounds, and other additives that influence ink properties. Common chemicals found in printing inks include 1-octane, 2-butanone, butyl acetate, citric acid, cyclohexanone, dichloromethane, ethyl acetate, ethylene glycol, gum arabic, isopropanol, methyl isobutyl ketone (used as dilution solvents), n-heptane, organic pigments, phthalate esters, polybutylene terephthalate resin, soybean oil, toluene, xylene, and polyvinyl acetate (used as adhesives and glues) (Tsai et al. 2016). Enhancements in ink adhesion and overall printing performance can be achieved through the incorporation of polyurethane-based polymers (Liu et al. 2022).

### Separation of cellulose fibres and their basic characterization

Waste paper cellulose is typically obtained through a combination of chemical and mechanical processes. The three most commonly used methods include pre-hydrolysis (using either alkali or mineral acid), alkaline pulping (typically with NaOH), and subsequent bleaching using hydrogen peroxide (H_2_O_2_) or sodium hypochlorite (NaOCl) (Hanafiah et al. 2019; Gunjan et al. 2023). These conventional processes are technically demanding; therefore, in our study, we opted for a simpler approach: releasing cellulose fibres by dissolving the filler material, PCC. Precipitated calcium carbonate, a synthetically produced filler commonly present in waste paper, exhibits properties distinct from those of natural calcite. It contains all three polymorphous modifications of calcium carbonate: calcite + aragonite + vaterite. Recent studies highlight PCC’s higher purity, controlled morphology, and surface modifiability, making it more suitable for industrial processing and the effective release of cellulose fibres (Kim et al. 2021). Given these differences, our research focused on identifying the most effective dissolution method to release the embedded cellulose fibres.

Mineralogical analysis of office paper (Table 1) confirms that the filler consists exclusively of various crystalline forms of calcium carbonate (CaCO₃), which are soluble in slightly acidic environments. The dissolution of calcium occurs according to the reactions described in Eqs. (1–4) Phipps and Lorusso (2001):

1 $$\;\begin{array}{cc}{\mathrm{CaCO}}_{3(\mathrm s)}\;\leftrightarrow\;\mathrm C{\mathrm a^{2+}}_{(\mathrm{aq})}\;+\;\mathrm C{\mathrm O_3^{2-}}_{(\mathrm{aq})}&{\mathrm K}_{\mathrm{so}}\;=\;\lbrack\mathrm{Ca}^{2+}\rbrack\lbrack\mathrm{CO}_3^{2-}\rbrack\end{array}$$

2 $$\;\begin{array}{cc}{\mathrm H^+}_{(\mathrm{aq})}\;+\;\mathrm C{\mathrm O_3^{2-}}_{(\mathrm{aq})}\;\leftrightarrow\;\mathrm{HC}{\mathrm O_3^-}_{(\mathrm{aq})}&{\mathrm K}_1\;=\;\lbrack\mathrm{HCO}_3^-\rbrack/\lbrack\mathrm{CO}_3^{2-}\rbrack\rbrack\lbrack\mathrm H^+\rbrack\end{array}$$

3 $$\;\begin{array}{cc}{\mathrm H^+}_{(\mathrm{aq})}\;+\;{\mathrm{HCO}_3^-}_{(\mathrm{aq})}\;\leftrightarrow\;{\mathrm{CO}}_{2(\mathrm{aq})}\;+\;{\mathrm H}_2{\mathrm O}_{(\mathrm l)}&{\mathrm K}_2\;=\;\lbrack{\mathrm{CO}}_2\rbrack/\lbrack\mathrm{HCO}_3^-\rbrack\lbrack\mathrm H^+\rbrack\end{array}$$

4 $$\;\begin{array}{cc}{\mathrm{CO}}_{2(\mathrm{aq})}\;\leftrightarrow\;{\mathrm{CO}}_{2(\mathrm g)}&{\mathrm K}_{\mathrm H}\;=\mathrm P({\mathrm{CO}}_2)/\lbrack{\mathrm{CO}}_2\rbrack\end{array}$$

The amount of calcium (Ca) extracted depends on several factors, including solvent type and concentration, particle size, and the solid-to-liquid (S/L) ratio. For paper sludge, Ca concentration in solution increased up to pH 5.5, where maximum leachability was observed, reaching 54% when using 0.7 M HCl or CH_3_COOH at an S/L ratio of 1:25 (Kim and Kim 2018).

**Fig. 2:**
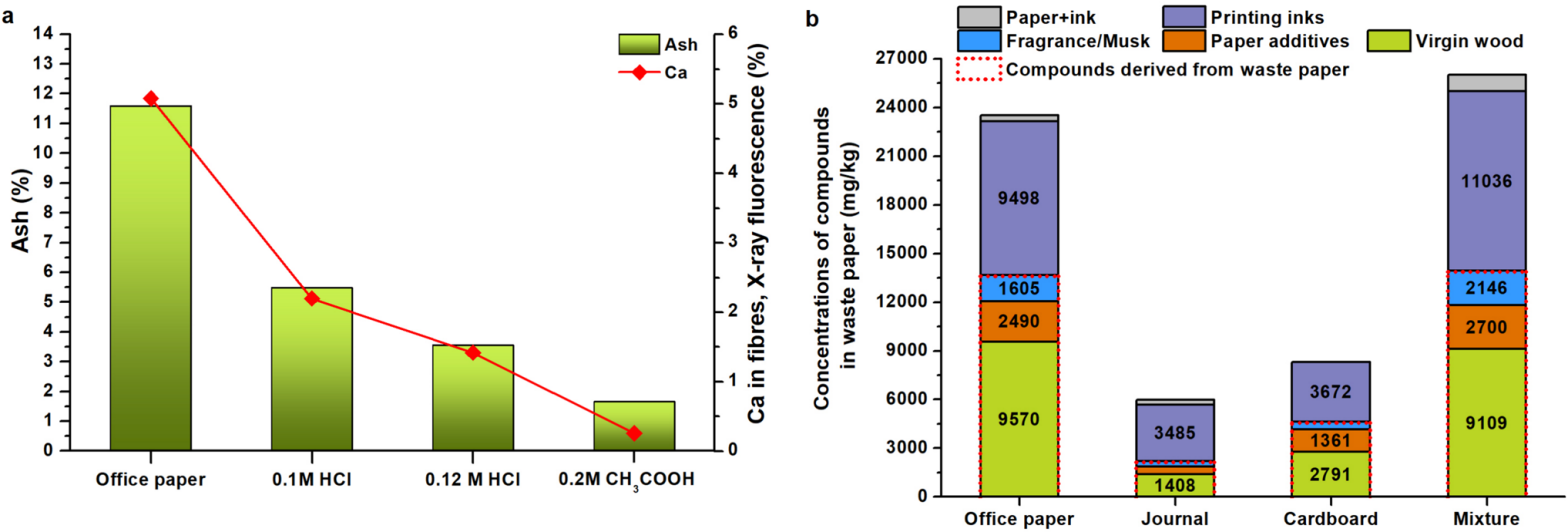
Amounts of ash in office paper and in cellulose fibres after dissolving PCC (**a**); compounds identified in waste paper (**b**)

In the case of office paper leaching, hydrochloric acid (HCl) concentrations above 0.12 M caused the solution pH to drop below 5. Similarly, when acetic acid was applied, pH fell below 5 at concentrations exceeding 0.2 M CH₃COOH. Acetic acid at 0.2 M demonstrated higher efficiency in carbonate removal, as evidenced by ash reduction and decreased Ca content in the embossed cellulose fibres (Fig. 2). At this concentration, Ca removal efficiency reached 95% and was further improved to 98.5% following subsequent washing.

The removal of carbonates to isolate pure cellulose fibres in an acidic medium is effective for office paper, cardboard, and mixed paper, where carbonates represent the primary filler component. The potential applications of separated cellulose are largely determined by its physicochemical properties, including crystallinity, purity, particle or fibre size, surface chemistry (presence of hydroxyl groups), thermal stability relevant for composite manufacturing, and its reactivity/modifiability enabling functionalization.

The fibre dimensions were influenced by the applied disintegration method. The lengths ranged from 0.88 to 5.93 mm, with an average of 2.87 ± 1.38 mm. The widths varied between 0.05 and 0.20 mm, with an average value of 0.11 ± 0.04 mm. One of the key parameters of cellulose is its degree of crystallinity, as it directly influences mechanical strength, thermal stability, and enzymatic accessibility—properties critical for industrial processing and material performance. In this study, two absorbance ratios derived from FTIR spectroscopy were used as indicators of crystalline and amorphous phase distribution: A₁₄₂₉/A₈₉₆ (Oh et al. 2005) and A₈₉₆/A₁₀₅₀ (Park et al. 2010). The band at 1429 cm⁻^1^ corresponds to CH₂ vibrations in crystalline structures, whereas the 896 cm⁻^1^ band represents C–H deformations in amorphous regions. Additionally, the band at 1050 cm⁻^1^ is associated with ordered, crystalline domains of cellulose. The measured FTIR absorbance ratios were A₁₄₂₉/A₈₉₆ = 0.995 and A₈₉₆/A₁₀₅₀ = 0.991, both suggesting a higher contribution of amorphous regions compared to crystalline phases in the analysed sample.

The separated cellulose obtained from waste paper, characterized by an enhanced amorphous structure as revealed by FTIR analysis, offers improved processability, ink dispersion, and substrate flexibility. These properties are highly desirable for 3D printing technologies, making the material suitable for biocomposite filaments, printable gels, functional bioinks, and even food packaging applications.

### Organic compounds in waste paper

The chemical compounds identified in waste paper were categorized into six groups based on their origin: virgin wood-derived compounds, papermaking additives, ink and paint compounds, ink and papermaking compounds, and unclassified substances. Among these groups, ink-derived compounds exhibited the highest concentrations in mixed paper and office paper samples, averaging 11.03 ± 0.57 g/kg.

A total of 138 chemical compounds were identified in waste paper samples using the TD-GC/MS method. These compounds were classified into four groups based on their origin: those derived from waste paper, printing inks, paper/printing inks (where the origin could not be distinguished), and unknown sources. Within the waste paper group, two subcategories were defined: virgin wood-derived compounds and additives used during the papermaking process to optimize paper properties, including fragrances from essential oils and synthetic musks. In terms of compound count, paper-derived substances were slightly more prevalent, accounting for 67 compounds compared to those originating from printing inks. Additionally, six compounds were found to originate from either paper or printing inks, potentially.

#### Chemical compounds coming from virgin wood

**Fig. 3:**
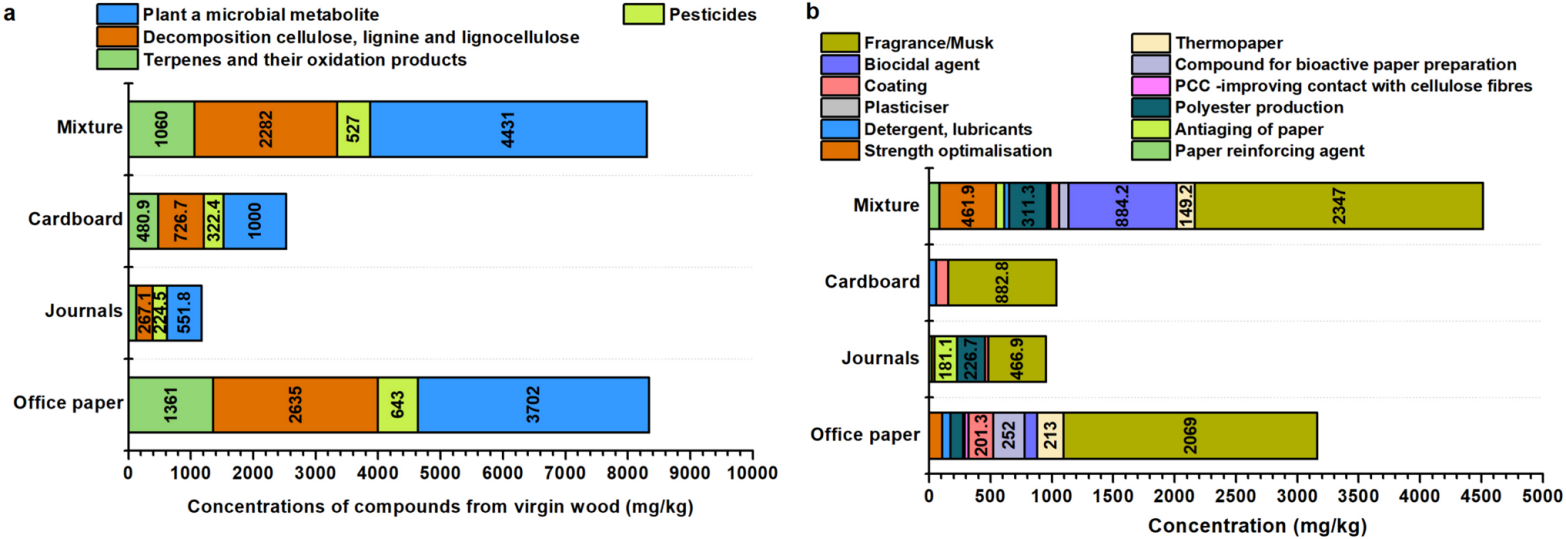
Identified amounts of chemical compounds originating from virgin wood (**a**); division of organic compounds identified in waste paper based on their function in the papermaking process (**b**)

A total of 31 chemical compounds originating from virgin wood were identified in waste paper, having not been removed during the production process. These compounds were divided into four categories, ranked by abundance: plant and microbial metabolites (14 compounds), decomposition products of cellulose and lignin (9 compounds), terpenes and their oxidation products (6 compounds), and pesticides (2 compounds) (Table 2 and Table S1,Fig. 3). The highest concentrations of virgin wood-derived compounds were found in office paper and mixed paper samples. The group of wood extractives includes a variety of wood resins comprising monoterpenes, resin acids, fatty acids, fatty alcohols, sterols, stearyl esters, and triglycerides (Dou et al. 2023). Terpenes and their oxidative products were quantified at 1361.25 ± 134.62 mg/kg in office paper and 126.98 ± 29.20 mg/kg in journal paper. Notably, (1S-endo)−1,7,7-trimethyl-bicyclo[2.2.1]heptan-2-ol and 2,6,6-trimethyl-bicyclo(3.1.1)heptane-2,3-diol—identified as major oxidation products of α-pinene—were also included in this group (Cheng et al. 2023). Among the cellulose and lignin decomposition products, coniferyl aldehyde was detected, a compound formed enzymatically from lignin in species such as *Picea abies* and *Pinus sylvestris* (Hänninen et al. 2011).

**Table 2:** Chemical compounds contained in virgin wood paper, including their classification according to GHS criteria

Group by origin	Chemical compound	Office papers		Journal		Cardboard		Mixture		GHS classification			"Stickies"
		mg/kg								HH	EH	Irritant	
		AVG	STD	AVG	STD	AVG	STD	AVG	STD				
Terpenes and their oxidation products	1-Methyl-4-(1-methylethyl)−1,3-cyclohexadiene			2.15	0.24	73.06	14.92	19.73	4.22	✓			
	1,3,3-Trimethyl- 2-oxabicyclo[2.2.2]octan-6-ol			55.95	8.54	182.21	37.27	472.33	87.69				
	5-Pentadecanone	255.26	52.20	57.69	9.37	214.94	22.56	71.67	22.16			✓	
	(1S-endo)−1,7,7-Trimethyl-bicyclo[2.2.1]heptan-2-ol	414.83	41.14	11.20	2.56	10.72	2.37	351.55	89.31				
	2,6,6-Trimethyl-bicyclo(3.1.1)heptane-2,3-diol	489.67	37.65					129.38	87.66				
	Exo-2-hydroxycineole	201.49	15.23					15.42	2.27				
Decomposition of cellulose, lignin, and lignocellulose	2-Ethyl-5-propylcyclopentanone			19.99	7.61			48.78	6.98				
	2,3,3,4-Tetramethyl-pentane					214.60	27.85	95.21	23.46			✓	
	2-Hexadecanone	401.20	31.33	116.47	20.15	345.45	49.54	115.54	33.78			✓	
	1-(4-Hydroxy-3-methoxyphenyl)−2-propanone	1194.49	523.20					202.47	52.27			✓	
	2-Methoxy-4-propylphenol							38.65	8.37				
	Coniferyl aldehyde	871.83	211.60					721.32	137.64				
	Levoglucosenone	167.13	37.98					878.25	214.35				
	Octadecane			28.60	4.39			46.54	12.55				
	Pentacosane			102.00	20.55	166.60	38.37	135.30	35.72				✓
Pesticides	1,2-Dihydro-3H-1,2,4-triazol-3-one	210.50	32.46					288.97	102.16	✓			
	3-Oxo-2-pentyl-cyclopentaneacetic acid methyl ester	432.54	27.24	224.48	27.36	322.43	34.36	238.04	74.33		✓		
Plant a microbial metabolite	2-Pentadecanone	412.61	17.56	129.29	41.33	295.47	31.37	152.83	32.64			✓	
	2′,4′-Dihydroxypropiophenone							24.15	4.27			✓	
	2,6-Pyridinedicarboxylic acid							9.47	32.33			✓	
	3-Ethyl-3-octanol	35.63	4.21			54.45	2.23	21.83	5.11				
	3-Undecanone			23.36	4.24	190.22	52.33	58.88	7.31				
	4,6′-Dimethoxy-2′-(tert.-butyldimethylsilyl)oxychalcone			10.08	1.56	23.25	4.61	30.43	6.14				
	2,4-Dihydroxy-6-methyl-benzaldehyde	2464.54	412.70					2237.22	567.42			✓	
	Benzoic acid pentyl ester	80.77	10.35	19.93	3.27			26.81	5.39				
	Heneicosane	131.29	22.57	50.65	8.56	113.12	22.37	82.73	11.13				✓
	Octacosane	189.62	39.41	69.30	24.21	144.14	17.12	157.19	21.54			✓	✓
	Allyl ethyl ester oxalic acid			77.51	26.86			1409.59	523.78				
	3-Hydroxy-2,2,4-trimethylpentyl ester 2-methyl-propanoic acid	184.09	37.65	52.59	11.37			77.50	22.84				
	Sulfurous acid, 2-ethylhexyl hexyl ester			27.02	6.54			20.65	4.39				
	Tetracosane	203.06	22.12	92.03	22.49	179.73	44.32	121.25	11.77			✓	✓

*AVG* average value, *EH* environmental hazard, *HH* health hazard, *STD *standard deviation

The growing demand for recycled paper as a substitute for virgin cellulose fibres presents challenges related to the presence of organic sticky contaminants, commonly referred to as “stickies.” Currently, recycled materials comprise up to 40% of paper production inputs (Ballinas-Casarrubias et al. 2020). The presence of stickies significantly affects the papermaking process, with concentrations in waste paper ranging between 3 and 5%. These contaminants primarily include polyvinyl acetate polymers, styrene-butadiene rubber (including butylated hydroxytoluene), siloxanes, polyamines such as dimethylamine and dimethylpropylamine, and a group of 13 paraffin waxes (Wang et al. 2023). Among these waxes, four compounds—heneicosane (C_21_H_44_), octacosane (C_28_H_58_), tetracosane (C_24_H_50_), and pentacosane (C_25_H_52_)—were consistently detected in all types of waste paper, with concentrations ranging from 50.6 to 203.1 mg/kg and an average value of 129.2 ± 45.1 mg/kg. These substances are also commonly found in wood extractives. The highest concentrations of stickies were observed in cardboard and office paper samples.

#### Chemical compounds from papermaking

A total of 19 compounds used to enhance paper properties and 15 fragrance-related components—including three musks—were identified in waste paper samples. All of these compounds were detected in mixed waste paper. In a journal paper, only five compounds were found, one of which (pentanoic acid) is associated with anti-ageing applications, along with four fragrance components. Office paper contained 13 functional compounds and 7 fragrance or musk-related substances, with a total concentration of 3,164.1 ± 620.6 mg/kg (Fig. 3). In cardboard samples, four property-enhancing compounds and five fragrance/musk components were identified. The highest concentrations in both cardboard and office paper were associated with octanoic and octadecanoic acids, which contribute to hydrophobicity and sizing performance.

Additional compounds identified in waste paper samples were found to enhance specific material properties. These include acrylamide, which contributes to paper reinforcement; methylene diacrylamide and ethylhydrazone acetaldehyde, used to optimize mechanical strength; and 2-propenenitrile and tetraethylsilane, which improve hydrophobic performance. Tributyl acetylcitrate was detected as a coating agent and plasticizer, while p-terphenyl and benzoic acid, 4-methylpent-2-yl ester were used to strengthen fibre interconnections and facilitate the integration of PCC. Modification of surface characteristics was associated with the presence of N,N-diethyl-4-methyl-benzamide (Table 3 and Table S2). Eugenol, identified in both paper mixtures and office paper, serves as a functional additive in the development of bioactive packaging materials (Muratore et al. 2020). To counteract odour-related effects generated during papermaking, biocidal agents such as pyrolo[3,2-d]pyrimidin-2,4(1H,3H)-dione and p-octylacetophenone were introduced. In addition, 11 fragrance compounds of biogenic origin and 3 synthetic musks—galaxolide, celestolide, and 6-hydroxy-6-methyl-bicyclo[3.3.0]octan-3-one were detected. Fragrances and musks accounted for the highest proportion of total additives across paper types: 65.4 ± 5.6% in office paper, 53.0 ± 4.2% in mixed paper, and up to 50.2 ± 10.1% in journal samples.

**Table 3:** Chemical compounds added to improve properties (additives) or the technological process of papermaking additives in paper, including their classification according to GHS criteria

Group of chemical compounds by use	Chemical compound	Office paper		Journals		Cardboard		Mixture		GHS classification		
		mg/kg								HH	EH	Irritant
		AVG	STD	AVG	STD	AVG	STD	AVG	STD			
Additives for improving paper properties	Acrylamide			21.02	2.11			85.63	45.69	✓		
	Methylenediacrylamide	56.41	5.47					60.02	2.55	✓		
	Ethylhydrazone acetaldehyde	49.50	4.34	25.02	5.37			401.86	210.85			
	2-Propenenitrile							96.76	11.55	✓		
	Tetraethyl-silane	72.69	11.88					62.14	7.46			
	Octadecanoic acid	536.01	212.72			324.28	33.82	190.54	174.20			✓
	Octanoic acid	789.27	154.87			723.01	111.67	210.65	116.68			
	Pentanoic acid			181.05	125.77			63.52	33.11			
	Methyl myristate	70.20	12.36			57.75	5.24	41.33	14.48			
	3-(Acetyloxy)propanoic acid anhydride	99.51	24.63					59.63	28.20			
	Hexahydro-1-methyl-2H-azepin-2-one			226.73	54.36			251.64	17.61			
	p-Terphenyl	13.40	4.81					8.52	3.45		✓	
	Benzoic acid, 4-methylpent-2-yl ester	34.28	2.55					14.63	3.89			
	N,N-Diethyl-4-methyl-benzamide							13.86	0.97			
	Tributyl acetylcitrate	201.31	41.81	30.21	5.55	96.32	15.39	60.37	14.58			
	Eugenol	252.03	33.42					72.48	26.96			
	Pyrolo[3,2-d]pyrimidin-2,4(1H,3H)-dione	105.68	17.79					877.22	245.56			
	p-Octylacetophenone							7.00	0.87			✓
	1-(Phenylmethoxy)naphthalene	212.96	41.63					149.19	45.09			✓
Fragrance—natural origin	1-(4-Hydroxy-3,5-dimethoxyphenyl)−1-propanone	86.07	6.27	47.75	3.22	117.33	55.54	75.72	28.74			✓
	1-Methyl-4-(1-methylethenyl)−1,2-cyclohexanediol	190.45	102.57					74.38	22.07			✓
	2-Methoxy-4-vinylphenol	295.29	104.11					527.93	164.50			✓
	3-Decanone							52.32	11.31			
	Dihydro-4-methyl-5-pentyl-2(3H)-furanone							112.06	24.55			
	Dihydro-5-pentyl-2(3H)-furanone			235.62	58.97			397.54	114.49			
	5-Heptyldihydro-2(3H)-furanone	464.23	49.44	147.52	23.84	398.50	113.39	208.22	50.84		✓	
	Acetyl valeryl							34.68	11.35			✓
	Delta-nonalactone	414.60	23.82			237.67	82.41	63.86	15.37			
	1,2-Cyclohexanedione					25.78	4.25	53.53	19.62			
	2-Methoxy-4-propylphenol					26.94	5.37	38.65	8.28			✓
	Trans-ocimenol							75.24	22.34			✓
Musks	8-Ethyl-4,6,6,8-tetramethyl-3,4,6,7-tetrahydro-1H-cyclopenta(G)−2-benzopyran	98.74	14.26	36.02	8.21	76.61	16.81	49.01	18.10			
	6-Hydroxy-6-methyl-bicyclo[3.3.0]octan-3-one	519.42	102.33					572.67	137.65			
	Celestolide							11.53	2.22			

*AVG* average value, *EH *environmental hazard, *HH* health hazard, *STD* standard deviation

Evidence of polyester (PES) fibre addition was confirmed by the detection of 3-(acetyloxy)propanoic acid anhydride and hexahydro-1-methyl-2H-azepin-2-one (m-methylcaprolactam), with peak concentrations found in waste paper derived from journals. Additionally, the presence of 1-(phenylmethoxy)naphthalene—a compound originating from thermal paper—was identified in both office and mixed paper samples (US EPA 2015).

#### Chemical compounds from printing inks

Varnishes composed of additives, solvents or diluents, oils, and resins, along with vehicles, are essential components that determine the functional properties of printing inks. These substances are responsible for effectively transferring pigment onto the substrate, securing its adhesion, and delivering key performance features such as printability, durability, and visual appearance. Today, over one million distinct ink formulations are in use across Europe (EuPIA 2023), reflecting the complexity and diversity of requirements within the printing industry.

**Fig. 4:**
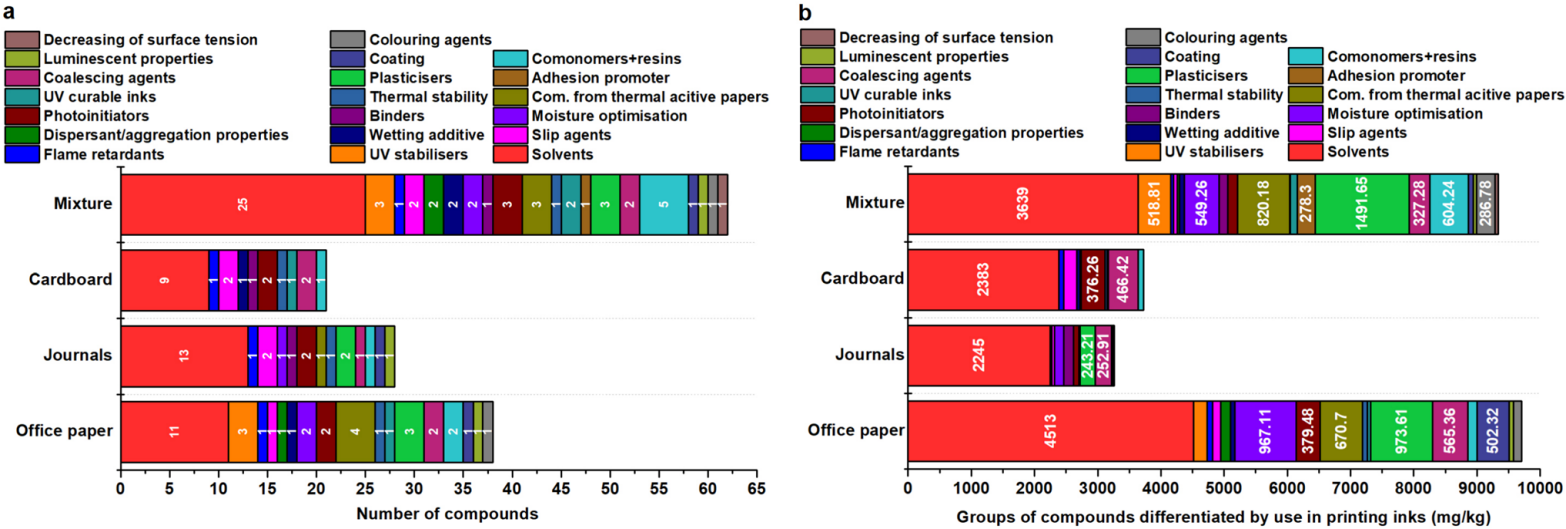
Classification of organic compounds identified in printing inks into groups according to their use (**a**); number of compounds identified in each group (**b**)

Solvents represent the largest group of chemical compounds found in waste paper, both in terms of diversity and total concentration (Fig. 4). A total of 25 solvent compounds were identified in mixed paper materials, followed by 13 in journal paper, 11 in office paper, and 9 in cardboard samples (Table 4 and Table S3).

**Table 4:** Chemical compounds utilized in printing inks (solvents and additives), including their classification according to GHS criteria

Chemical compound	Office papers		Journals		Cardboard		Mixture		GHS classification		
	mg/kg								HH	EH	Irritant
	AVG	STD	AVG	STD	AVG	STD	AVG	STD			
t-Butyl isobutyl ketone	126.66	70.40					26.99	4.83			
1,1,1-Trifluoro-2-butanone			65.95	4.88			70.21	3.01			✓
1,2-Dimethoxy-ethane			30.89	2.53			52.63	15.37	✓		
1,4-Butanediol			496.32	250.40			142.20	22.38			✓
3-Methyl-2-butanone							45.65	12.29			
2-Ethyl-1-hexanol			30.83	14.33			48.21	8.66			✓
2-Phenoxy-ethanol	753.31	305.10					321.84	84.29		✓	
2,6-Diisopropylnaphthalene	240.72	102.33	85.79	42.12			47.45	3.55		✓	
3-Methyl-3-buten-2-one							20.31	8.32			
4-Tert-octylphenol					0.96	0.22	6.54	3.94		✓	
5-Ethyl-2-methyl-heptane							43.12	4.78			
6-Undecanone			59.26	10.54			69.68	7.17		✓	
N-[(Dimethylamino)methylidene]-acetamide					40.15	5.21	72.24	22.69			
Benzoic acid, 2-ethylhexyl ester	154.80	45.85	55.14	8.39	139.97	42.88	90.19	22.34	✓		
4-Hydroxy-α,α,4-trimethyl-cyclohexanemethanol	458.71	83.26	458.34	104.01	363.49		286.48	43.92		✓	
Dibutyl phthalate	22.52	2.93	209.77	52.38	437.11	170.57	260.95	56.24	✓		
Diethyl phthalate	503.96	55.57	400.20	81.37	427.54	27.68	498.65	51.74		✓	
Diethylene glycol			145.21	33.32			110.29	24.70		✓	
Dodecanoic acid	640.49	273.80			485.84	114.51	108.23	23.65		✓	
2-(2-Butoxyethoxy) ethanol acetate	398.34	133.03					210.20	87.99		✓	
n-Hexadecanoic acid	740.59	118.62	178.85	25.93	405.16	102.32	369.86	233.53		✓	
Isobutyl benzoate			28.69	4.05	82.78	15.07	47.36	27.48			
N,N-Dimethylacetamide							352.11	57.51	✓		
DL-2,3-butanediol							123.52	47.36			
Propylene carbonate	472.43	182.37					214.52	64.33			
2,5-Bis(1,1-dimethylethyl)−1,4-benzenediol	87.17	8.13					63.21	16.94		✓	
2-Methoxy-4-(1-propenyl)-phenol	114.45	13.54					430.28	223.32			
Butylated hydroxytoluene	12.02	4.46					25.32	9.40		✓	
1-Chloro-2-propanol phosphate (3:1)	85.65	10.32	25.97	2.30	80.57	14.37	38.36	29.92			✓
Isopropyl myristate	129.78	56.80	31.48	7.55	134.23		35.96	8.49			✓
Isopropyl palmitate			14.48	5.37	67.38	28.02	24.91	7.77			✓
1-Butanamine							1468.70	516.91			✓
Resorcinol	158.53	105.37					9.52	1.52		✓	
Pyrrolidine							23.51	5.33			
Diethylene glycol			145.21	42.38			110.29	24.70			✓
2-Hexyl-1-decanol							59.63	11.13			
Methyl tetradecanoate	70.20	25.23			57.75	17.77	21.62	8.74			✓
2-Ethylhexanoic acid	275.01	105.68					258.37	11.76			
2-Propanamine			160.64	76.55	16.73	3.23	133.93	34.95			✓
2,3-Dihydro-1,1,3-trimethyl-3-phenyl-1H-indene			16.92	3.34			13.42	2.48			✓
Dodecyl acrylate	140.64	54.84	70.88	6.56	174.61	88.91	61.10	5.76		✓	
2-Ethylhexyl- 2 metylbenzoate	238.84	80.38			201.65	102.44	84.79	11.64			
1-(Phenylmethoxy)-naphthalene	212.96	41.37					149.19	45.09			
1,1′-[1,2-ethanediylbis(oxy)]bisbenzene	133.40	28.55					486.84	249.92		✓	
1-Dodecanol	309.44	88.60					184.15	23.55		✓	
Bisphenol S	14.89	7.98	3.60	1.05					✓		
1,2-Ethanediol monobenzoate	74.50	30.33	9.21	4.66	30.69	8.56	11.18	1.33			✓
N,N′-Methylenebis-2-propenamide	56.41	8.34					60.02	2.55	✓		
Benzophenone					23.39	8.01	53.78	21.49	✓		
2-Pyrrolidinone							278.30	55.68	✓		
1,3-Diacetin			11.55	2.07			18.09	4.62			
Bis(2-ethylhexyl) phthalate	118.83								✓		
Bisphenol A	6.73								✓		
Hexanedioic acid, dioctyl ester							53,96	29,44			✓
Nonanoic acid	848.05	196.50	231.66	89.92			1419.61	594.11			✓
Benzoic acid, undecyl ester	87.47	33.56			93.47	14.42	32.57	7.33			
Methyl hexadecanoate	477.88	98.93	252.91	102.14	372.96	103.78	294.71	82.55			✓
1,3,5-Triazine-2,4(1H,3H)-dione					93.05	42.81	32.51	3.18			✓
4-Methyl-1,3-isobenzofurandione	28.91	3.30					32.52	2.55			✓
Phthalic anhydride							460.48	104.55	✓		
5-(1,1-Dimethylethyl)−1,3-benzenedicarboxylic acid							33.94	17.31			✓
Isothiocyanatocyclohexane	114.93	48.03	23.03	5.88			44.78	3.36	✓		
Methylphosphonic acid 2TMS derivative	502.32	266.55	8.91	1.90			80.80	15.89			
n-Decanoic acid	692.10	284.44	142.23	29.34			290.90	36.66			✓
Diethyl(decyloxy)borane	74.73		13.30	2.56			52.86	31.14			
(6-Isopropyl-3,4-bis(methylamino)−2,4,6-cycloheptatrienylidene)malononitrile	129.79						286.78	111.01			
(4aS-trans)−1,2,3,4,4a,9,10,10a-octahydro-1,1,4a-trimethyl-7-(1-methylethyl)phenanthrene (Abieta-8,11,13-triene)							55.63	23.84		✓	

*AVG* average value, *EH* environmental hazard, *HH* health hazard, *STD* standard deviation

Although the mixed paper contained the highest number of solvent-related compounds, the overall concentrations of these substances were greater in office paper. Specifically, 46.4% of all identified compounds in office paper were attributed to printing ink. In mixed paper, printing ink-related compounds accounted for 47.8%, while journals contained 46.7%. Cardboard had the lowest proportion of such compounds (34.5%), reflecting its comparatively smaller printed surface area.

The highest concentrations of solvent compounds varied depending on paper type. In the office paper, 2-phenoxyethanol and n-hexadecanoic acid were most abundant, while the journal paper exhibited peak levels of 1,4-butanediol and 4-hydroxy-α,α,4-trimethyl-cyclohexanemethanol. Cardboard samples showed elevated concentrations of dodecanoic acid and dibutyl phthalate, both commonly used in printing inks and as plasticizers. In mixed paper material, the dominant compounds were n-hexanoic acid and diethyl phthalate. Furthermore, three compounds linked to thermal paper were explicitly identified in mixed paper samples. This type of waste paper also contained five compounds associated with comonomers or resin systems, forming the second-largest numerical group. The third group, comprising four substances, was related to particle dispersion and aggregation behaviours. In addition, five plasticizers—bis(2-ethylhexyl) phthalate (DEHP), bisphenol A (BPA), hexanedioic acid, dioctyl ester, and nonanoic acid—along with three photoinitiator compounds, were detected in the samples.

A distinct group of six compounds was identified that are commonly used in both papermaking and printing ink formulation (Table 5 and Table S4). This group includes four plasticizers: two phthalate-based (diisobutyl phthalate and dimethyl phthalate) and two non-phthalate alternatives—2,2,4-trimethyl-1,3-pentanediol diisobutyrate (KODAFLEX TXIB) and bis(2-ethylhexyl) terephthalate (DEHT). In addition, two solvent compounds were detected: 2-phenoxyethanol and 2-butanone, which serve functional roles in both production processes.

**Table 5:** Chemical compounds utilized for paper making and also for printing inks, including their classification according to GHS criteria

Chemical compound	Office paper		Journals		Cardboard		Mixture		GHS classification		
	mg/kg								HH	EH	Irritant
	AVG	STD	AVG	STD	AVG	STD	AVG	STD			
2-Phenoxyethanol	753.31	247.85			427.55	77.37	321.84	112.35			✓
2-Butanone			20.72	4.22			23.65	2.44			✓
2,2,4-Trimethyl-1,3-pentanediol diisobutyrate	374.15	82.13	213.85	44.79	84.16	5.56	172.25	18.93	✓		
Bis(2-ethylhexyl) terephthalate			8.54	1.88			11.96	2.42			
Diisobutyl phthalate			15.23	3.12			647.91	184.39	✓		
Dimethyl phthalate			21.36	4.24			169.53	44.47			✓

*AVG* average value, *EH* environmental hazard, *HH* health hazard, *STD* standard deviation

### Assessment of potential hazards of identified compounds

**Fig. 5:**
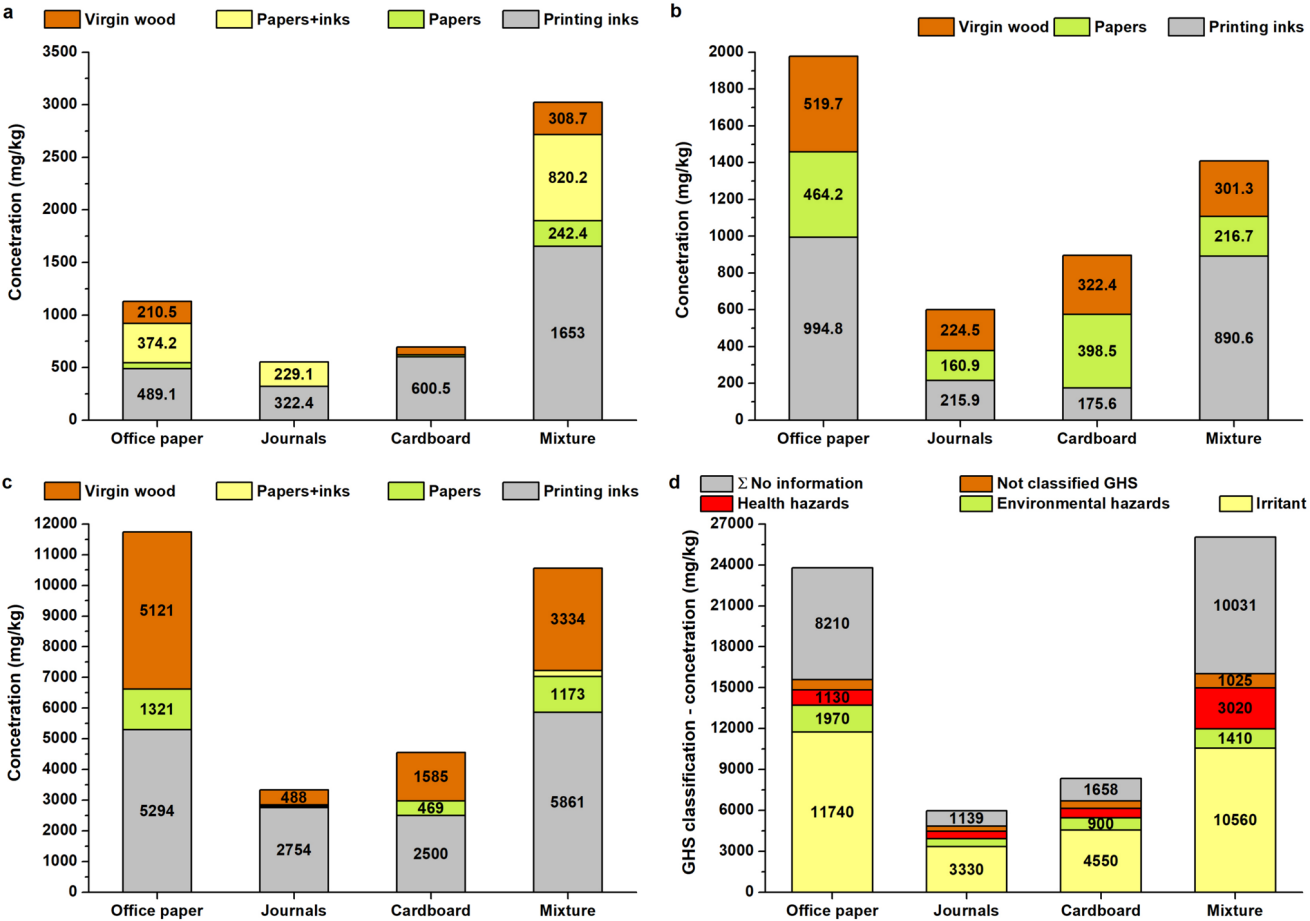
Division of compounds using GHS classification for each paper type (**a**, **b**, and **c**); the total distribution of compounds identified by TD-GC/MS according to GHS classification (**d**)

The highest concentrations of compounds classified as health hazards (HH) were identified in mixed paper material, up to three times higher than in office paper (Fig. 5). A majority of HH compounds (12) originated from printing inks. Among them were persistent organic pollutants such as bisphenol A (BPA) (Cimmino et al. 2020), di(2-ethylhexyl)phthalate (DEHP) (Chen et al. 2023), and benzophenone – the latter commonly used as a UV filter (ECHA 2018). BPA is primarily employed in thermochromic inks for thermal printing paper, where concentrations can reach up to 16.3 g/kg. In contrast, its levels in office paper are significantly lower, around 280 mg/kg, and only 0.8 mg/kg in journal-derived waste paper (Pivnenko et al. 2016). BPA concentration ranges between 126 and 460 mg/kg have also been reported (Vinković et al. 2023), though in the present study, BPA was detected exclusively in office paper at low concentrations of 6.72 ± 1.2 mg/kg. Due to its potential health risks, the use of BPA as a plasticizer is subject to regulatory scrutiny. In 2024, the European Commission proposed a ban on the use of bisphenol A in plastics intended for food contact. The draft regulation would strictly prohibit BPA in manufacturing food-contact plastics, varnishes, coatings, printing inks, and related industrial applications.

In office paper, phthalate plasticizer DEHP was identified at concentrations of 118.8 ± 24.6 mg/kg, which corresponds to the concentrations reported for paper recycling (10.0–63.3 mg/kg). Cardboard contains relatively lower concentrations of 10.1–70.5 mg/kg. The highest concentrations are reported for packaging (57.7–393.3 mg/kg) and disposable diapers (14.2–322.2 mg/kg) (U.S. Department of Health And Human Services 2022). Printing inks also contain other phthalate plasticizers, dibutyl phthalate (DiBP), which can reach concentrations of up to 120 mg/kg in waste paper (Pivnenko et al. 2015). The DiBP concentrations in the analysed waste paper vary in a large range from 22.5 ± 3.7 mg/kg in office paper through 209.8 ± 23.7 mg/kg in journals to 437.1 ± 134.5 mg/kg in cardboard. Benzophenone was found in cardboard (23.4 ± 2.7 mg/kg) and mixed paper material (53.8 ± 7.9 mg/kg). Comparative information is only available for cardboard packaging materials, where the concentrations of benzophenone ranged from 20 to 330 mg/m^2^ (Liu And Mabury 2021).

Due to its potential health risks, the use of BPA as a plasticizer is subject to regulatory scrutiny. In 2024, the European Commission proposed a ban on the use of bisphenol A in plastics intended for food contact. The draft regulation would strictly prohibit BPA in manufacturing food-contact plastics, varnishes, coatings, printing inks, and related industrial applications.

Undifferentiated compounds from paper or printing inks containing KODAFLEX TXIB and DiBP also show high concentrations of agents with HH. The highest concentrations of DiBP, up to 437 ± 38 mg/kg, were found in cardboard. The lowest concentrations in office paper were 22.5 ± 9.7 mg/kg. The highest concentrations of DiBP in cardboard were also confirmed by Pivnenko et al. (2015). They report 3 mg/kg for office paper and 32 mg/kg for cardboard, which is due to the addition of recycled paper. There is a significant difference between our values and concentrations, as reported by Pivnenko et al. (2015). The values are difficult to compare because waste paper contains a variety of compounds as additives; data on their presence is sparse.

The proportion of health hazard (HH) compounds originating from virgin wood was found to be significant (Fig. 5). Among the prevailing substances were 1,2-dihydro-3H-1,2,4-triazol-3-one (a pesticide) and 3-oxo-2-pentyl-cyclopentaneacetic acid methyl ester (an insecticide). Their presence aligns with findings reported by Růžičková et al. (2021), confirming the persistence of agrochemical residues in virgin wood. In mixed paper materials, HH compounds were most prevalent among substances derived from printing inks, particularly solvents. These included UV-curable ink constituents such as N,N′-methylenebis-2-propenamide and benzophenone, as well as the adhesion-promoting compound 2-pyrrolidinone. Additional contributors to HH classification were comonomers and resins, including phthalic anhydride, isothiocyanatocyclohexane, and various polyurethane resins. Available data on benzophenone concentrations in waste paper are limited. Liu and Mabury (2021) reported levels of 11.5 mg/kg in food packaging media. In the present study, benzophenone was detected exclusively in mixed paper and cardboard samples, with concentrations of 53.8 mg/kg and 23.4 mg/kg, respectively.

The highest concentrations of compounds classified as environmental hazards (EH) were identified in office paper, primarily originating from printing inks. In virgin wood, EH-classified substances include pesticides such as p-terphenyl and 5-heptyldihydro-2(3H)-furanone, which were also detected in journal and cardboard samples. EH compounds used in printing inks encompass various solvents, including 2,6-diisopropylnaphthalene (DINP), 4-tert-octylphenol, 6-undecanone, butylated hydroxytoluene (BHT), and 2,5-bis(1,1-dimethylethyl)−1,4-benzenediol—as well as the dispersant resorcinol. In thermal recording paper, inks commonly contain ethylene glycol diphenyl ether and 1-dodecanol, while 6-hydroxy-6-methyl-bicyclo[3.3.0]octan-3-one is used to reduce surface tension. BHT, a widely employed synthetic phenolic antioxidant, is used across numerous product categories; approximately 6% of global BHT production is designated for printing ink applications. Concentrations of BHT in food packaging materials range from 0.24 to 0.95 mg/kg, whereas magazine waste paper shows levels between 0.040 and 0.37 mg/kg (Liu And Mabury 2021). These values are nearly tenfold lower than the concentrations observed in office paper. Elevated levels of DINP are associated with the use of recycled paper, where DINP replaced polychlorinated biphenyls previously used in carbonless copy paper dyes (Coltro et al. 2021). In the present study, DINP concentrations reached 240 ± 24 mg/kg in office paper, while journal-derived waste samples exhibited significantly lower levels of 86 ± 17 mg/kg.

The highest concentrations among compounds monitored under the Globally Harmonized System (GHS) were associated with irritants, reaching approximately 12 g/kg. These substances were proportionally distributed between those originating from printing inks and virgin wood. Virgin wood contributed four compounds formed during cellulose thermal degradation, such as 2-ethyl-5-propylcyclopentanone (Ojha And Vinu 2015), and additional compounds resulting from lignin depolymerization, including 2-methoxy-4-propylphenol (Zhang et al. 2018). Within the plant and microbial metabolite group, seven compounds demonstrated irritant properties; for example, 2-pentanone, which also functions as a biocidal agent (Tyśkiewicz et al. 2019). A total of 25 irritant compounds were linked to printing inks. Among these, nine were solvents and three were polymeric resins. Each of the three categories (slip agents, plasticizers, and dispersants) contained two compounds. The remaining compounds were distributed across other ink-related functional groups, including photoinitiators, thermal stability agents, coalescing agents, anti-evaporation agents, photoionization compounds, binders, and wetting agents—each represented by a single substance.

Nineteen compounds classified as health hazards (HH) were identified in waste paper. The highest number of HH compounds (16) was found in mixed paper materials, which also exhibited the greatest concentrations. Office paper contained ten HH compounds, journals eight, and cardboard the fewest. Among all HH substances, phthalic anhydride had the highest concentrations in mixed paper, reaching 460.5 ± 75.5 mg/kg.

In terms of environmental hazards (EH), 12 compounds were identified across the samples. All were present in mixed paper material, nine in office paper, and only six in cardboard. Journal waste exhibited the lowest concentrations of EH compounds. As with HH substances, most EH compounds originate from printing inks. However, pesticides derived from virgin wood also contribute significantly to EH classifications in waste paper. The presence and concentrations of HH and EH compounds play a crucial role in determining the feasibility of further utilizing cellulose fibres obtained from recycled paper.

### Effect of removal of precipitated calcium carbonate from office paper on the quality of cellulose fibres

Pure cellulose fibre extraction was performed using 0.2 M acetic acid (CH_3_COOH), as detailed in the “Materials and their pre-treatment for further analysis” section. This procedure applies only to office paper, where PCC is the sole filler. Organic compounds were analysed via the TD-GC/MS method, as in previous sections. Following PCC removal, a total of 31 compounds were identified in the office paper sample (Table 6). Eight compounds were identified both in cellulose fibres and in the original waste paper. Removed amounts of compounds ranged between 4 and 90% (Table 6). An additional ten compounds were newly detected exclusively in the cellulose fibres. Their possible formation is described in Table S5. Furthermore, 13 compounds were probably formed through mutual interactions between detected substances or as a result of acetic acid–mediated transformations during the leaching process. The newly identified compounds found in the cellulose fibres are presumed to have originated from polymer coatings, adhesives, or surface treatments, where they were chemically bound or incorporated within the paper matrix. Upon leaching with acetic acid, these layers underwent degradation, releasing the compounds. Owing to their polar and reactive nature, the compounds then interacted with cellulose and became detectable within the fibre matrix, even though they had not been directly identified in the original waste paper.

**Table 6:** Chemical compounds in office paper determined after extraction with acetic acid (OP-CH_3_COOH) and in original waste office paper (OP) in mg/kg

Matrix	Chemical compounds	CAS	OP-CH_3_COOH	STD	Waste paper	Removed	Origin/Use	GHS classification			
			mg/kg	mg/kg	mg/kg	%		HH	EH	I	AT
Paper/ink	3,5,5-Trimethyl-hexanoyl chloride	36727–29-4	761.75	112.41			Production of esterified cellulose nanofibres				✓
	4-Methoxy-1,3-benzenediamine	615-05−4	49.52	6.54			Acrylic fibres (nylon, polyester)	✓			
Virgin wood	1-(1H-imidazol-4-yl)−1-pentanone	69393-15−3	28.86	14.12			Alkaloid				
	1-Methyl-4-(1-methylethyl)−1,3-cyclohexadiene^*^	99-86−5	7.21	1.22	73.06	90.1	Component of terpenoid, α-terpinene	✓			
	1H-Naphtho[2,1-b]pyran, 3-ethenyldodecahydro-3,4a,7,7,10a-pentamethyl-, [3S-(3.alpha.,4a.alpha.,6a.beta.,10a.alpha.,10b.beta.)]-		3.05	0.56			Essential oil compounds with antioxidant activities				
	1,2,4-Triazine-3,5(2H,4H)-dione*	461-89−2	34.07	7.23	35.51	4.04	Metabolite of the herbicide metribuzin				
	4-Methyl-2-hexanone	105-42−0	33.00	17.24			Natural substances and extractives			✓	
	1,3,3-Trimethyl-2-oxabicyclo[2.2.2]octan-6-ol^*^	18679-48−6	716.57	241.50	1082	33.8	Biooxidation of cineole				
	1,3,3-Trimethyl-2-oxabicyclo[2.2.2]octan-6-ol, acetate	72257-53−5	142.00	23.14			Formed through acetylation				
	5-Acetyldihydro-2(3H)-furanone	29393-32−6	203.01	27.29			Natural substances and extractives			✓	
	Hexacosane	630-01−3	90.03	14.32			Natural products—compounds of "Stickies"			✓	
	Tricosane	638-67−5	54.25	17.38			Natural products—compounds of "Stickies"				
Paper	1,2-Ethanediol diformate	629-15−2	71.09	16.27			pH regulating agent				
	Table 2	137-89−3	47.84	21.25			Plasticiser for paper and cardboard products. Replacement of DEHP	✓			
	1,6-Dioxacyclododecane-7,12-dione	777-95−7	15.98	4.32			Production of polyurethane, adhesives in multilayer packaging materials			✓	
	2-Oxooctanoic acid	328-51−8	816.17	156.15			Surface tension			✓	
	N,N′-Methylenebis-2-propenamide^*^	110-26−9	11.51	1.27	60.02	80.8	Paper making	✓			
	Dihydro-5-pentyl-2(3H)-furanone^*^	104-61−0	359.08	82.33	754.35	52.4	Fragrance mostly for cellulose cardboard				
	5-Heptyldihydro-2(3H)-furanone^*^	104-67−6	174.60	33.12	408.22	57.2	Fragrance mostly for cellulose cardboard		✓		
	5-Hexyldihydro-2(3H)-furanone*	706-14−9	222.05	47.54	298.50	25.6	Fragrance				
	Dicyandiamide	461-58−5	73.36	18.26			Improvement in ink absorption, flame retardant in the paper industry			✓	
	Octadecanal	638-66−4	120.66	21.97			Biologically active pheromones			✓	
	Sec-butyl acetate	105-46−4	47.87	23.47			Paper processing, solvent for nitrocellulose lacquers				
Printing inks	1,3-Cyclohexanedione*	504-02−9	9.91	0.86	53.53	81.5	Decomposition product of resorcinol, a polymeric dispersant for ink jet			✓	
	2-Ethyl-1-butanol	97-95−0	26.70	13.14			Solvent for printing inks			✓	
	3-Butene-1,2-diol	497-06−3	250.06	51.97			The major metabolite of 1,3-butadiene, used for the ink			✓	
	3-Heptanone	106-35−4	354.09	52.29			Solvent for paint			✓	
	Bis(2-(dimethylamino)ethyl) ether	3033-62−3	103.10	22.86			UV printing ingredients				✓
	Cyclopentane	287-92−3	34.72	12.21			Non-polar solvent				
	Ethyl cyanoacetate	105-56−6	25.05	4.27			Ethyl acetate, a solvent for resins in inks			✓	
	2-Acetyl-resorcinol	699-83−2	51.02	11.11			Produced by the chemical reaction of acetic acid			✓	
Σ All compounds								116	175	2070	864

^*^Designation of chemicals identified in the original waste paper, *AT* acute toxicity, *EH* environmental hazard, *HH* health hazard, *I* irritant, *OP* office paper, *STD* standard deviation

Following the removal of PCC, enhanced access to cellulose fibres enabled the identification of eight compounds derived from virgin wood and eight compounds originating from printing inks. Two of the ink-related compounds—1,3-cyclohexanedione and 2-acetyl-resorcinol—were formed through the conversion of resorcinol, originally present in the untreated office paper. Four additional substances were identified as solvents. Bis(2-(dimethylamino)ethyl) ether was detected as an additive specific to UV-printing applications. Another compound, 3-butene-1,2-diol, represents the primary degradation product of 1,3-butadiene (BD), used in ink resin formulations. Furthermore, eight newly identified compounds in OP–CH_3_COOH were associated with various functional roles in paper production (Table 6). Two additional compounds were found to originate either from printing inks or paper materials. The first, 4-methoxy-1,3-benzenediamine, is employed in both ink production and the synthesis of polyester fibres, which may serve as alternatives to natural fibres. The second, 3,5,5-trimethylhexanoyl chloride, functions as a plasticizer and is also utilized in the production of esterified cellulose nanofibres.

**Fig. 6:**
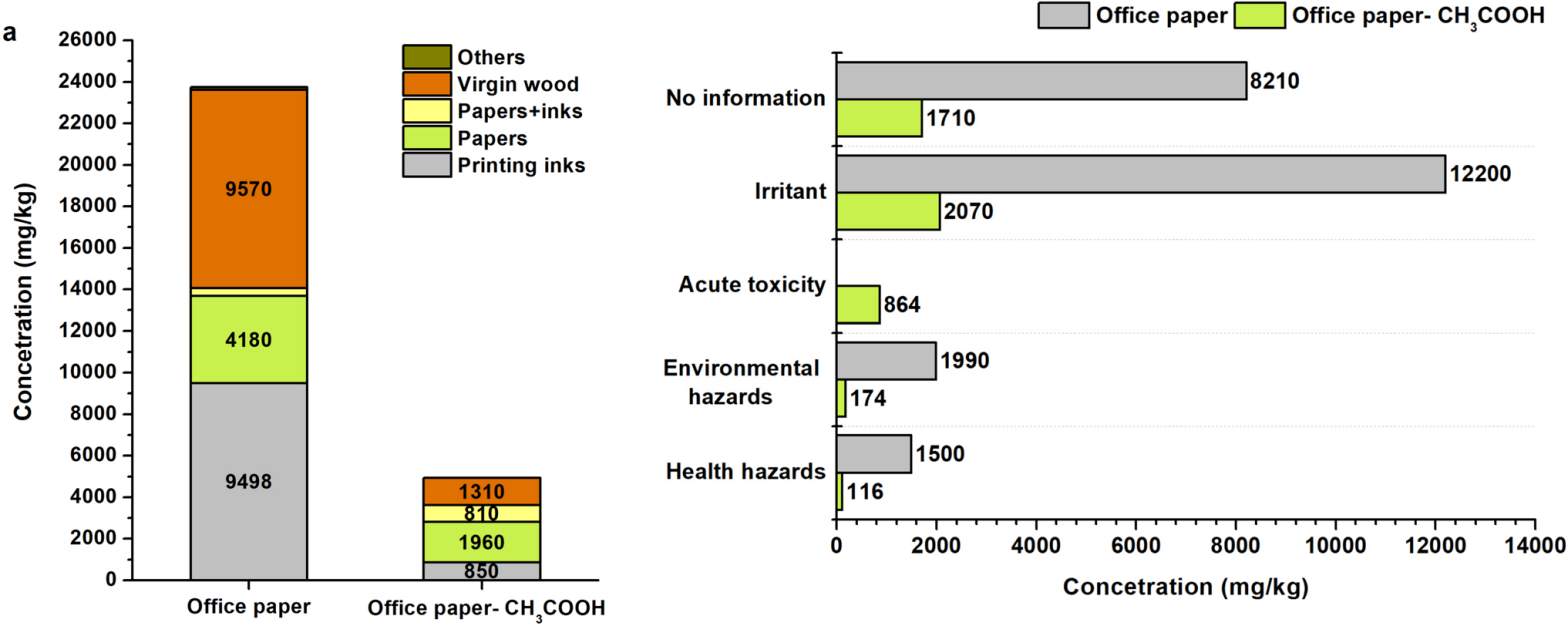
Comparison of concentrations of identified compounds in waste office paper and office paper (OP) extracted in CH_3_COOH (**a**); decrease in concentrations of hazardous and not identified compounds after extraction in CH_3_COOH (**b**)

**Fig. 7:**
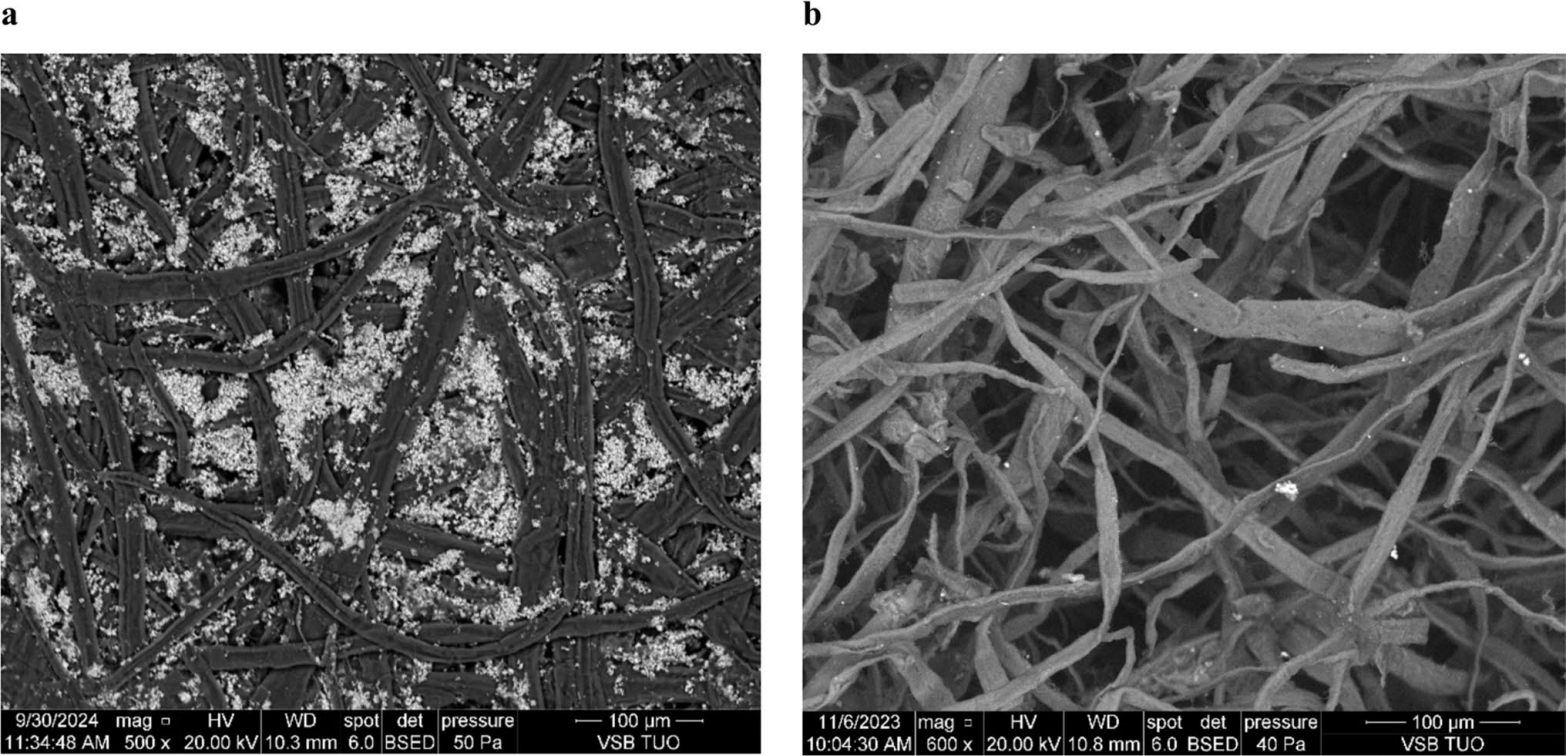
Cellulose fibres before (**a**) and after extraction (**b**) with CH_3_COOH and washing with distilled water in the scanning electron microscope

According to the Globally Harmonized System (GHS) classification, 4 compounds identified in the waste paper samples fall under health hazards (HH), 1 compound is categorized as an environmental hazard (EH), 2 exhibit acute toxicity, and 13 are classified as irritants. Figure 6 illustrates a 79% reduction in the total concentration of identified compounds between untreated office paper (OP) and acetic acid-treated samples (OP–CH_3_COOH), including a significant decrease in GHS-classified hazardous substances. Additionally, as shown in Table 6, no persistent organic pollutants were detected in OP–CH_3_COOH. These results demonstrate that extraction using 0.2 M acetic acid followed by washing yields a highly purified cellulose fibre product, suitable for further applications (Fig. 7).

The newly identified compounds were released from the cellulose matrix during acid extraction and subsequently detected using the TD-GC/MS method. Although thermal desorption is generally expected to release most compounds during TD-GC/MS analysis, its reliability diminishes when analytes are structurally embedded within the cellulose. Thermal decomposition of cellulose occurs at temperatures ranging from 440 to 580 °C under oxidative conditions and 280–380 °C in an inert atmosphere (Shen et al. 2013), both of which exceed the operational temperature of the thermal desorption unit used in the analytical setup.

## Conclusion

Following acetic acid (CH₃COOH) treatment, 31 of the original 138 compounds identified in waste office paper remained embedded in the cellulose fibres. The purpose of this study was to document the presence of natural organic substances derived from virgin wood that persist despite conventional paper manufacturing processes. These compounds constitute up to 40.4 ± 5.8% of the total organic content in office paper. Notably, approximately 39% of virgin wood-derived compounds were classified as “stickies”, which can interfere with paper recycling operations. The extraction procedure using CH₃COOH, followed by thorough washing, resulted in a 92% reduction in compounds classified as health hazards (HH). This includes the effective removal of persistent organic pollutants such as di(2-ethylhexyl)phthalate (DEHP), bisphenol A (BPA), and benzophenone. Additionally, compounds categorized as environmental hazards (EH) were reduced by 92.6%, irritants by 83%, and substances with unknown hazard classifications by 80%. These findings demonstrate that the removal of precipitated calcium carbonate enables the recovery of highly purified cellulose fibres from office paper, suitable for further technological applications. The chemical data indicate significantly reduced levels of additives (contaminants) following fibre separation. Nonetheless, to confirm the safety of these fibres for food-contact applications, additional experimental validation is required, particularly through migration testing and assessments of functional performance.

Moreover, our approach avoids the use of stronger corrosive acids or chlorine-based bleaching agents, which pose greater risks to workers and the environment. A preliminary risk assessment suggests that, with proper process design, acetic acid treatment can be safely implemented at scale, while offering improved material purity and reduced chemical hazards compared to conventional deinking and extraction processes.

## Supplementary Information

Below is the link to the electronic supplementary material.

Supplementary file 1 (DOCX 45.4 KB)

## Data Availability

All data generated or analysed during this study are included in this published article.

## Abbreviations

AT: Acute toxicity
BD: 1,3-Butadiene
BHT: Butylated hydroxytoluene
BPA: Bisphenol A
CIS: Cooled injection system
CMC: Carboxymethyl cellulose
CNs: Cellulose nanocrystals
DEHP: Bis(2-ethylhexyl) phthalate
DEHT: Bis(2-ethylhexyl) terephthalate
DiBP: Dibutyl phthalate
DINP: 2,6-Diisopropylnaphthalene
DOIP: Bis(2-ethylhexyl) ester 1,3-benzenedicarboxylic acid
EH: Environmental hazards
EuPIA: European Printing Ink Association
GHS: Globally Harmonized System of Classification and Labelling of Chemicals
HH: Health hazards
I: Irritant
KODAFLEX TXIB: 2,2,4-Trimethyl-1,3-pentanediol diisobutyrate
OP: Office paper
PAC: Polyaluminium chloride
PCC: Precipitated calcium carbonate
PES: Polyester
PPCs: Plant fibre/plastic composites
STD: Standard deviation
stickies: Organic sticky contaminants
US EPA: United States Environmental Protection Agency
UV: Ultraviolet
