# Supplementary material for: Removal of fillers and chemical reagents from waste paper for its sustainable use
Source: Environ Sci Pollut Res Int. 2025 Nov 7;32(45):25730–53. doi: 10.1007/s11356-025-37133-5 (PMC12662857; doi:10.1007/s11356-025-37133-5)
Supplement: Supplementary file 1 — (DOCX 45.4 KB) [file 11356_2025_37133_MOESM1_ESM.docx]

**Removal of fillers and chemical reagents from waste paper for its sustainable use**

Marek Kucbel^a,*^, Helena Raclavská^a^, Jana Růžičková^a^, Michal Šafář^a^, Pavel Kantor^a^, Karolina Slamová^b^, Jarmila Drozdová^c^

^a^ VSB–Technical University of Ostrava, CEET/ENET Centre, 17. listopadu 15/2172, 708 00 Ostrava-Poruba, Czech Republic (marek.kucbel@vsb.cz; helena.raclavska@vsb.cz; jana.ruzickova@vsb.cz; michal.safar@vsb.cz; pavel.kantor@vsb.cz)

^b^ VSB–Technical University of Ostrava, Institute of Foreign Languages, 17. listopadu 15/2172, 708 00 Ostrava-Poruba, Czech Republic (karolina.slamova@vsb.cz)

^c^ Institute of Technology and Business in České Budějovice, Faculty of Technology, Department of Mechanical Engineering, Okružní 517/10, 370 01 České Budějovice, Czech Republic (drozdova@mail.vstecb.cz)

** Corresponding author: Marek Kucbel, E-mail: marek.kucbel@vsb.cz, phone: +420 596 995 448*

**Table S1** Chemical compounds contained in virgin wood paper, including their classification according to GHS criteria.

| **Group by origin** | **Chemical compound** | **Synonym** | **CAS** | **Chemical formula** | **Origin/utilisation** |
| --- | --- | --- | --- | --- | --- |
| Terpenes and their oxidation products | 1-methyl-4-(1-methylethyl)-1,3-cyclohexadiene |  | 99-86-5 | C_10_H_16_ | Component of terpenoid, α-terpinen, foliar insecticide |
|  | 1,3,3-trimethyl- 2-oxabicyclo[2.2.2]octan-6-ol | exo-2-Hydroxycineole | 18679-48-6 | C_10_H_18_O_2_ | Biooxidation of cineole |
|  | 5-pentadecanone |  | 92862-23-2 | C_15_H_30_O | Compound of essential oils with antibacterial effect |
|  | (1S-endo)-1,7,7-trimethyl-bicyclo[2.2.1]heptan-2-ol |  | 124-76-5 | C_10_H_18_O | Major oxidation products of a-pinene, fragrance |
|  | 2,6,6-trimethyl-bicyclo(3.1.1)heptane-2,3-diol |  | 53404-49-2 | C_10_H_18_O_2_ | Major oxidation products of a-pinene |
|  | Exo-2-hydroxycineole |  | 18679-48-6 | C_10_H_18_O_2_ | Extractives, compounds of essential oil |
| Decomposition of cellulose, lignin and lignocellulose | 2-ethyl-5-propylcyclopentanone |  | 38468-47-2 | C_10_H_18_O | Cellulose decomposition |
|  | 2,3,3,4-tetramethyl-pentane |  | 1186-53-4 | C_9_H_20_ | Cellulose decomposition |
|  | 2-hexadecanone |  | 18787-63-8 | C_16_H_32_O | Depolymerisation of lignin |
|  | 1-(4-hydroxy-3-methoxyphenyl)-2-propanone | Quiacylacetone | 2503-46-0 | C_10_H_12_O_3_ | Depolymerisation of lignin |
|  | 2-methoxy-4-propylphenol |  | 2785-87-7 | C_10_H_14_O_2_ | Decomposition of lignin |
|  | Coniferyl aldehyde |  | 458-36-6 | C_10_H_10_O_3_ | Decomposition of lignin |
|  | Levoglucosenone |  | 37112-31-5 | C_6_H_6_O_3_ | Decomposition of saccharides and lignin |
|  | Octadecane |  | 593-45-3 | C_18_H_38_ | Decomposition of lignocellulose |
|  | Pentacosane |  | 629-99-2 | C_25_H_52_ | Decomposition of lignocellulose |
| Pesticides | 1,2-dihydro-3H-1,2,4-triazol-3-one |  | 930-33-6 | C_2_H_3_N_3_O | Pesticide transformation products |
|  | 3-oxo-2-pentyl-cyclopentaneacetic acid methyl ester |  | 39924-52-2 | C_13_H_20_O_3_ | Pesticide/insecticide |
| Plant a microbial metabolite | 2-pentadecanone |  | 2345-28-0 | C_15_H_30_O | Plant secondary metabolites with bioactive effects |
|  | 2',4'-dihydroxypropiophenone | Paroxypropione | 5792-36-9 | C_9_H_10_O_3_ | Natural phenylpropanoid found in the herbs of *Cedrus deodara.* |
|  | 2,6-pyridinedicarboxylic acid | Dipicolinic acid | 499-83-2 | C_7_H_5_NO_4_ | Metabolite produced by many bacterial and fungal species. |
|  | 3-ethyl-3-octanol |  | 2051-32-3 | C_10_H_22_O | Plant metabolite, fragrance agent |
|  | 3-undecanone |  | 2216-87-7 | C_11_H_22_O | Plant metabolite, ketonization of carboxylic acids |
|  | 4,6'-dimethoxy-2'-(tert.-butyldimethylsilyl)oxychalcone |  |  |  | Derive from unsaturated fatty acids (linolenic acid) or by oxidation of carotenoids such as lycopene and phytoene. |
|  | 2,4-dihydroxy-6-methyl-benzaldehyde |  | 487-69-4 | C_8_H_8_O_3_ | Natural product of plants, fungal metabolite |
|  | Benzoic acid pentyl ester |  | 2049-96-9 | C_12_H_16_O_2_ | Derivatives of the major building blocks benzoyl/benzyl in various natural plant products. |
|  | Heneicosane |  | 629-94-7 | C_21_H_44_ | Plant metabolite, Candy wrappers based on plastic and paper materials |
|  | Octacosane |  | 630-02-4 | C_28_H_58_ | Plant metabolite with antibacterial activity |
|  | Allyl ethyl ester oxalic acid |  |  | C_7_H_10_O_4_ | Plant tissue, metabolite |
|  | 3-hydroxy-2,2,4-trimethylpentyl ester 2-methyl-propanoic acid |  | 74367-34-3 | C_12_H_24_O_3_ | Plant metabolite |
|  | Sulfurous acid, 2-ethylhexyl hexyl ester |  | 959067-41-5 | C_14_H_30_O_3_S | Produced by salt stress through biosynthesis |
|  | Tetracosane |  | 646-31-1 | C_24_H_50_ | Plant metabolite and volatile oil component |

**Table S2** Chemical compounds added to improve properties (additives) or the technological process of papermaking additives in paper, including their classification according to GHS criteria.

| **Group of chemical compounds by use** | **Chemical compound** | **Synonym** | **CAS** | **Chemical formula** | **Origin/utilisation** |
| --- | --- | --- | --- | --- | --- |
| Additives for  improving  paper  properties | Acrylamide |  | 79-06-1 | C_3_H_5_NO | Paper reinforcing agent |
|  | Methylenediacrylamide |  | 110-26-9 | C_7_H_10_N_2_O_2_ | Strength optimisation |
|  | Ethylhydrazone acetaldehyde |  |  | C_4_H_10_N_2_ |  |
|  | 2-propenenitrile |  | 107-13-1 | C_3_H_3_N | Hydrophobic properties, sizing |
|  | Tetraethyl-silane |  | 631-36-7 | C_8_H_20_Si |  |
|  | Octadecanoic acid | Stearic acid | 57-11-4 | C_18_H_36_O_2_ |  |
|  | Octanoic acid | Caprylic acid | 124-07-2 | C_8_H_16_O_2_ |  |
|  | Pentanoic acid | Valeric acid | 109-52-4 | C_5_H_10_O_2_ | Anti-ageing of paper |
|  | Methyl myristate |  | 124-10-7 | C_15_H_30_O_2_ | Detergent, lubricants |
|  | 3-(acetyloxy)propanoic acid anhydride |  | 55656-58-1 | C_10_H_14_O_7_ | Polyester production |
|  | Hexahydro-1-methyl-2H-azepin-2-one | N-methylcaprolactam | 2556-73-2 | C_7_H_13_NO |  |
|  | p-terphenyl |  | 92-94-4 | C_18_H_14_ | Plasticiser |
|  | Benzoic acid, 4-methylpent-2-yl ester |  |  | C_13_H_18_O_2_ | PCC -improving contact with cellulose fibres |
|  | N,N-diethyl-4-methyl-benzamide | N,N-diethyl-p-toluamide | 2728-05-4 | C_12_H_17_NO | Coating |
|  | Tributyl acetylcitrate |  | 77-90-7 | C_20_H_34_O_8_ |  |
|  | Eugenol |  | 97-53-0 | C_10_H_12_O_2_ | Compound for bioactive paper preparation |
|  | Pyrolo[3,2-d]pyrimidin-2,4(1H,3H)-dione |  | 65996-50-1 | C_6_H_5_N_3_O_2_ | Biocidal agent |
|  | p-Octylacetophenone |  | 10541-56-7 | C_16_H_24_O |  |
|  | 1-(phenylmethoxy)naphthalene |  | 607-58-9 | C_17_H_14_O | Thermopaper |
| Fragrance –  natural  origin | 1-(4-hydroxy-3,5-dimethoxyphenyl)-1-propanone | Propiosyringone | 5650-43-1 | C_11_H_14_O_4_ | Eucalypt oil |
|  | 1-methyl-4-(1-methylethenyl)-1,2-cyclohexanediol | P-mentha-8(9)-en-1,2-diol | 57457-97-3 | C_10_H_18_O_2_ | Fragrance |
|  | 2-methoxy-4-vinylphenol |  | 7786-61-0 | C_9_H_10_O_2_ | Fragrance |
|  | 3-decanone |  | 928-80-3 | C_10_H_20_O | Secondary metabolite, fragrance |
|  | Dihydro-4-methyl-5-pentyl-2(3H)-furanone |  | 33673-62-0 | C_10_H_18_O_2_ | Fragrance - white tee |
|  | Dihydro-5-pentyl-2(3H)-furanone | Gamma nonalactone | 104-61-0 | C_9_H_16_O_2_ | Fragrance for cardboard |
|  | 5-heptyldihydro-2(3H)-furanone |  | 104-67-6 | C_11_H_20_O_2_ | Fragrance |
|  | Acetyl valeryl | 2,3-heptanedione | 96-04-8 | C_7_H_12_O_2_ | Fragrance |
|  | Delta-nonalactone |  | 3301-94-8 | C_9_H_16_O_2_ | Fragrance |
|  | 1,2-cyclohexanedione |  | 765-87-7 | C_6_H_8_O_2_ | Fragrance |
|  | 2-methoxy-4-propylphenol |  | 2785-87-7 | C_10_H_14_O_2_ | Fragrance, decomposition products of lignin |
|  | Trans-ocimenol |  | 7643-60-9 | C_10_H_16_O | Fragrance |
| Musks | 8-Ethyl-4,6,6,8-tetramethyl-3,4,6,7-tetrahydro-1H-cyclopenta(G)-2-benzopyran | Galaxolide, HHCB | 78448-49-4 | C_18_H_26_O | Synthetic musk, used for papermaking |
|  | 6-hydroxy-6-methyl-bicyclo[3.3.0]octan-3-one |  |  | C_9_H_14_O_2_ | Synthetic musk |
|  | Celestolide | ADBI | 13171-00-1 | C_17_H_24_O | Group of acetophenone, synthetic musk |

**Table S3** Chemical compounds utilised in printing inks (solvents and additives), including their classification according to GHS criteria.

| **Chemical compound** | **Synonym/abbreviation** | **CAS** | **Chemical formula** | **Origin/utilisation** |
| --- | --- | --- | --- | --- |
| t-Butyl isobutyl ketone |  | 14705-50-1 | C_9_H_18_O | Solvent |
| 1,1,1-trifluoro-2-butanone |  | 381-88-4 | C_4_H_5_F_3_O | Solvents |
| 1,2-dimethoxy-ethane |  | 110-71-4 | C_4_H_10_O_2_ | Non-polar solvent |
| 1,4-butanediol |  | 110-63-4 | C_4_H_10_O_2_ | Solvent |
| 3-methyl-2-butanone |  | 563-80-4 | C_5_H_10_O | Solvent |
| 2-ethyl-1-hexanol |  | 104-76-7 | C_8_H_18_O | Solvent |
| 2-phenoxy-ethanol |  | 122-99-6 | C_8_H_10_O_2_ | Solvent for cellulose acetate, inks, and resins |
| 2,6-Diisopropylnaphthalene | DINP | 24157-81-1 | C_16_H_20_ | Dyes in carbonless copy paper, solvent |
| 3-methyl-3-buten-2-one |  | 814-78-8 | C_5_H_8_O | Solvent for acrylic resins |
| 4-tert-octylphenol |  | 140-66-9 | C_14_H_22_O | Solvent + nonionic surfactants |
| 5-ethyl-2-methyl-heptane |  | 13475-78-0 | C_10_H_22_ | Solvent |
| 6-undecanone |  | 927-49-1 | C_11_H_22_O | Solvent |
| N-[(dimethylamino)methylidene]-acetamide |  | 38221-31-7 | C_5_H_10_N_2_O | Solvent |
| Benzoic acid, 2-ethylhexyl ester | EHBA | 5444-75-7 | C_15_H_22_O_2_ | Solvent |
| 4-hydroxy-α,α,4-trimethyl-cyclohexanemethanol | Terpin | 80-53-5 | C_7_H_14_O | Solvent |
| Dibutyl phthalate | DBP | 84-74-2 | C_16_H_22_O_4_ | Solvent |
| Diethyl Phthalate | DEP | 84-66-2 | C_12_H_14_O_4_ | Solvent |
| Diethylene glycol |  | 111-46-6 | C_4_H_10_O_3_ | Solvent |
| Dodecanoic acid |  | 143-07-7 | C_12_H_24_O | Solvent |
| 2-(2-butoxyethoxy) ethanol acetate |  | 124-17-4 | C_10_H_20_O_4_ | Solvent |
| n-Hexadecanoic acid | Palmitic acid | 57-10-3 | C_16_H_32_O_2_ | Solvent |
| Isobutyl benzoate | Benzoic acid, 2-methylpropyl ester | 120-50-3 | C_11_H_14_O_2_ | Solvent |
| N,N-dimethylacetamide |  | 127-19-5 | C_4_H_9_NO | Solvent |
| DL-2,3-butanediol |  | 513-85-9 | C_4_H_10_O_2_ | Solvent |
| Propylene carbonate |  | 108-32-7 | C_4_H_6_O_3_ | Solvent |
| 2,5-bis(1,1-dimethylethyl)-1,4-benzenediol | Naugard 451, DTBHQ | 88-58-4 | C_14_H_22_O_2_ | US stabilisers, antioxidants |
| 2-methoxy-4-(1-propenyl)-phenol | Isoeugenol | 97-54-1 | C_10_H_12_O_2_ |  |
| Butylated hydroxytoluene | BHT | 128-37-0 | C_15_H_24_O |  |
| 1-chloro-2-propanol phosphate (3:1) |  | 13674-84-5 | C_9_H_18_Cl_3_O_4_P | Flame retardant |
| Isopropyl myristate |  | 110-27-0 | C_17_H_34_O_2_ | Slip agents |
| Isopropyl palmitate |  | 142-91-6 | C_19_H_38_O_2_ |  |
| 1-butanamine |  | 109-73-9 | C_4_H_11_N | Printing ink, silicone elastomer |
| Resorcinol |  | 108-46-3 | C_6_H_6_O_2_ | Dispersant |
| Pyrrolidine |  | 123-75-1 | C_4_H_9_N | Dispersant and anticlogging agent |
| Diethylene glycol |  | 111-46-6 | C_4_H_10_O_3_ | Aggregation of dye molecules |
| 2-hexyl-1-decanol |  | 2425-77-6 | C_16_H_34_O | Moistening additive |
| Methyl tetradecanoate |  | 124-10-7 | C_15_H_30_O_2_ | Wetting agents |
| 2-ethylhexanoic acid |  | 149-57-5 | C_8_H_16_O_2_ | Used as driers for odourless inks |
| 2-propanamine |  | 75-31-0 | C_3_H_9_N | Binders |
| 2,3-dihydro-1,1,3-trimethyl-3-phenyl-1H-indene |  | 3910-35-8 | C_18_H_20_ | Photoinitiators |
| Dodecyl acrylate |  | 2156-97-0 | C_19_H_28_O_2_ |  |
| 2-ethylhexyl- 2 metylbenzoate |  |  | C_16_H_24_O_2_ |  |
| 1-(phenylmethoxy)-naphthalene |  | 607-58-9 | C_17_H_14_O | Thermal recording paper |
| 1,1'-[1,2-ethanediylbis(oxy)]bisbenzene | Ethylene glycol diphenyl ether | 104-66-5 | C_14_H_14_O_2_ |  |
| 1-dodecanol |  | 112-53-8 | C_12_H_26_O |  |
| Bisphenol S |  | 80-09-1 | C_12_H_10_O_4_S | Curing agent in thermopaper |
| 1,2-ethanediol monobenzoate | Ethylene glycol | 94-33-7 | C_9_H_10_O_3_ | Thermal stability of ink |
| N,N'-methylenebis-2-propenamide |  | 110-26-9 | C_7_H_10_N_2_O_2_ | UV curable inks |
| Benzophenone |  | 119-61-9 | C_13_H_10_O |  |
| 2-pyrrolidinone |  | 616-45-5 | C_4_H_7_NO | Adhesion enhancement |
| 1,3-diacetin | Glyceryl 1,3 diacetate | 105-70-4 | C_7_H_12_O_5_ | Plasticisers |
| Bis(2-ethylhexyl)phthalate | DEHP | 117-81-7 | C_24_H_38_O_4_ |  |
| Bisphenol A | BPA | 80-05-7 | C_15_H_16_O_2_ |  |
| Hexanedioic acid, dioctyl ester | Di-n-octyl adipate | 123-79-5 | C_22_H_42_O_4_ |  |
| Nonanoic acid | Biogennic+F133:Q133 | 112-05-0 | C_9_H_18_O_2_ |  |
| Benzoic acid, undecyl ester |  | 6316-30-9 | C_18_H_28_O_2_ | Coalescing agent for printing inks |
| Methyl ester hexadecanoic acid | Methyl palmitate | 112-39-0 | C_17_H_34_O_2_ |  |
| 1,3,5-triazine-2,4(1H,3H)-dione | Azuracil | 71-33-0 | C_3_H_3_N_3_O_2_ | Polymeric resins /monomers/precursors |
| 4-methyl-1,3-isobenzofurandione | 3-Methylphthalic anhydride | 57110-29-9 | C_9_H_12_O_3_ | Crosslinking comonomer for epoxy resins |
| Phthalic anhydride |  | 85-44-9 | C_8_H_4_O_3_ | Comonomer for epoxy resins and colourants |
| 5-(1,1-dimethylethyl)-1,3-benzenedicarboxylic acid | Terephthalic acids | 2359-09-3 | C_12_H_14_O_4_ | Plastic additives for dyes. Comonomer for the production of polyester |
| Isothiocyanatocyclohexane |  | 1122-82-3 | C_7_H_11_NS | Degradation products of polyurethane |
| Methylphosphonic acid 2TMS derivative |  | 18279-83-9 | C_7_H_21_O_3_PSi_2_ | Coating of metal pigments |
| n-Decanoic acid | Capric acids | 334-48-5 | C_10_H_20_O_2_ | Anti-evaporative layers |
| Diethyl(decyloxy)borane |  |  | C_14_H_31_BO | Luminescence + antibacterial agent |
| (6-Isopropyl-3,4-bis(methylamino)-2,4,6-cycloheptatrienylidene)malononitrile |  | 14203-76-0 | C_15_H_18_N_4_ | Magenta colouring agent - malonitrile |
| (4aS-trans)-1,2,3,4,4a,9,10,10a-octahydro-1,1,4a-trimethyl-7-(1-methylethyl)phenanthrene (Abieta-8,11,13-triene) |  | 85-01-8 | C_14_H_10_ | Decreasing surface tension |

**Table S4** Chemical compounds utilised for paper making and also for printing inks, including their classification according to GHS criteria.

| **Chemical compound** | **Synonym/abbreviation** | **CAS** | **Chemical formula** | **Origin/utilisation** |
| --- | --- | --- | --- | --- |
| 2-phenoxyethanol |  | 122-99-6 | C_8_H_10_O_2_ | Solvent in paints, in paper acetate cellulose |
| 2-butanone |  | 78-93-3 | C_4_H_8_O | Solvent in paints, transparent paper |
| 2,2,4-trimethyl-1,3-pentanediol diisobutyrate | KODAFLEX TXIB | 6846-50-0 | C_16_H_30_O_4_ | Plasticisers for printing inks and paper |
| Bis(2-ethylhexyl) terephthalate | DEHT | 6422-86-2 | C_24_H_38_O_4_ | Plasticisers for printing ink and paper, highest concentration in wet cotton wipes |
| Diisobutyl phthalate | DIBP | 84-69-5 | C_16_H_22_O_4_ | Plasticiser in dispersion glues and printing inks. Applied in paper and packaging for food |
| Dimethyl phthalate | DMP | 131-11-3 | C_10_H_10_O_4_ | Printing inks, paper coatings |

**Table S5** Newly identified compounds in cellulose fibres after acetic acid extraction and their presumed origin and formation mechanisms.

| **Compounds present in cellulose fibres after extraction with CH₃COOH** | **Chemical compounds** | **Mechanism of formation or transformation** |
| --- | --- | --- |
| Newly discovered compounds not previously found in waste paper | 1-(1H-imidazol-4-yl)-1-pentanone | 1-(1H-imidazol-4-yl)-1-pentanone was most likely released from technical additives present in the paper or formed as a degradation or condensation product during the leaching process. Due to its polar and reactive nature, it bonded to the cellulose fibres, making it detectable in the fibre matrix, even though it was not directly identified in the original paper. |
|  | 1,6-dioxacyclododecane-7,12-dione | 1,6-Dioxacyclododecane-7,12-dione may have been part of polymer coatings, adhesives, or surface treatments, where it was chemically bound or embedded within the matrix. During acetic acid leaching, these layers broke down, releasing the compound, which subsequently bonded to the cellulose. |
|  | 3-heptanone | 3-heptanone may have originated from adhesives, dyes, or surface treatments used in the paper, which were broken down during the acetic acid leaching process. In the original paper, it could have been bound within the matrix or present in concentrations too low to be detected. Thanks to its polar nature and ability to form hydrogen bonds, 3-heptanone could have selectively adsorbed onto the hydroxyl groups of cellulose, making it analytically detectable in the fibre material. |
|  | 3,5,5-trimethyl-hexanoyl chloride | 3,5,5-trimethylhexanoyl chloride was likely released from technical additives present in the paper during the leaching process. Due to its high reactivity toward hydroxyl groups, it subsequently bonded to the cellulose, making it detectable within the fibres even though it had not been identified in the original paper. |
|  | Bis(2-(dimethylamino)ethyl) ether | Bis(2-(dimethylamino)ethyl)ether was likely released from technical additives present in the paper or formed from chemical precursors during the leaching process. Owing to its polar nature, it bonded to the cellulose, making it detectable within the fibre matrix—even though it was not directly identified in the original paper. |
|  | Cyclopentane | Cyclopentane may have been part of polymer coatings, adhesives, or dyes, where it was chemically bound or entrapped within the matrix. During acetic acid leaching, these layers broke down, releasing the cyclopentane, which then bonded to the cellulose. |
|  | Dicyandiamide | Due to its high affinity for polar compounds, cellulose may have selectively bound DCDA, even if only trace amounts were originally present in the paper. During the leaching process, DCDA could have been released from polymer-based surface layers that were not detectable in the untreated material. |
|  | Ethyl cyanoacetate (ECA) | ECA may have been part of adhesives, inks, polymer coatings, or stabilisers that were bound within the paper matrix. During the acetic acid leaching process, these layers underwent degradation or hydrolysis, leading to the release of ECA, which subsequently bonded to the cellulose. |
|  | Hexacosane | Hexacosane and tricosane are commonly used in paper surface treatments through waxing, as well as in printing inks and adhesives. Acetic acid (0.2 M) is capable of disrupting interactions between cellulose fibres and hydrophobic compounds, facilitating their release or migration into the solution during leaching. |
|  | Tricosane |  |
| Newly formed compounds | 1H-naphtho[2,1-b]pyran, 3-ethenyldodecahydro-3,4a,7,7,10a-pentamethyl-, [3S-(3.alpha.,4a.alpha.,6a.beta.,10a.alpha.,10b.beta.)]- | It forms through the dimerisation of naphthopyrans (derived from terpenes) in acetic acid. |
|  | 1,2-ethanediol, diformate | In an acidic environment, diethylene glycol (DEG), which was identified in the paper, can undergo partial cleavage into ethylene oxide or ethylene(glycol) fragments. These then react with formic acid → leading to the formation of 1,2-ethanediol diformate. |
|  | Bis(2-ethylhexyl) ester 1,3-benzenedicarboxylic acid, DOIP | Bis(2-ethylhexyl) ester 1,3-benzenedicarboxylic acid (DOIP) could have formed during acetic acid leaching, which acts as a catalyst for esterification. Given that 2-ethylhexanol was confirmed to be present in the waste paper, the reaction with 1,3-benzenedicarboxylic acid, 5-(1,1-dimethylethyl)- is chemically plausible under the mildly acidic conditions, resulting in the formation of DOIP, which was subsequently detected in the cellulose fibre fraction. |
|  | 1,3,3-trimethyl-2-oxabicyclo[2.2.2]octan-6-ol, acetate | It forms through acetylation of terpene alcohols. |
|  | 2-acetyl-resorcinol | Resorcinol may have originally been bound within the paper or its surface treatments. During extraction with acetic acid, it was released and subsequently acetylated, producing acetylresorcinol, which has a higher affinity for cellulose and therefore became bound to the fibres. |
|  | 2-ethyl-1-butanol | Butyl alcohol can be formed from the degradation of butanediol through UV radiation or microbial activity. |
|  | 2-oxooctanoic acid | The compound originated from the UV-assisted oxidation of octanoic acid, identified in the waste paper matrix. |
|  | 3-Butene-1,2-diol | 3-methyl-3-buten-2-one (commonly known as methyl vinyl ketone) and 3-butene-1,2-diol are structurally related compounds, making their interconversion chemically feasible. Under conditions involving water, acetic acid, and possibly exposure to UV radiation or microbial activity, two key transformations may occur: hydration across the carbon–carbon double bond, leading to the formation of a diol structure; and reduction of the carbonyl group, resulting in the conversion of the ketone to an alcohol moiety. |
|  | 4-methyl-2-hexanone | Given the mild acidity (0.2 M), acetic acid is capable of mobilising low-boiling ketones and hydrophobic organics without significant cellulose damage. As 4-methyl-2-hexanone is commonly used as an industrial solvent or intermediate, its identification points to chemical residues derived from manufacturing or printing processes, selectively recovered in the acid phase. |
|  | 4-methoxy-1,3-benzenediamine | In acetic acid, hydrolysis or reduction of other aromatic compounds may occur — if nitro compounds or azo compounds were present, they could be converted into benzenediamine derivatives. |
|  | 5-acetyldihydro-2(3H)-furanone | The formation of 5-acetyldihydro-2(3H)-furanone from dihydro-5-pentyl-2(3H)-furanone could theoretically occur through acetylation at the C-5 position, provided that acetyl donors are present in the environment or that acetyl groups are released from the breakdown of other components (e.g., esters or side reactions involving acetic acid). |
|  | Octadecanal | The biotransformation of fatty acids, specifically octanoic acid identified in the waste paper |
|  | Sec-butyl acetate | Esterification of sec-butyl alcohol with acetic acid |
